# Climate variability modulates western US ozone air quality in spring via deep stratospheric intrusions

**DOI:** 10.1038/ncomms8105

**Published:** 2015-05-12

**Authors:** Meiyun Lin, Arlene M. Fiore, Larry W. Horowitz, Andrew O. Langford, Samuel J. Oltmans, David Tarasick, Harald E. Rieder

**Affiliations:** 1 Program in Atmospheric and Oceanic Sciences, Princeton University, Princeton, New Jersey 08540, USA; 2 NOAA Geophysical Fluid Dynamics Laboratory, Princeton, New Jersey 08540, USA; 3 Department of Earth and Environmental Sciences, Columbia University, New York City, New York 10027, USA; 4 Lamont-Doherty Earth-Observatory, Columbia University, Palisades, New York 10964, USA; 5 NOAA Earth System Research Laboratory, Boulder, Colorado 80305, USA; 6 Cooperative Institute for Research in Environmental Sciences, University of Colorado, Boulder, Colorado 80305, USA; 7 Experimental Studies Research Division, Meteorological Service of Canada, Environment Canada, Ontario, Canada M3H5T4; 8 Wegener Center for Climate and Global Change and IGAM/Institute of Physics, University of Graz, Graz 8010, Austria

## Abstract

Evidence suggests deep stratospheric intrusions can elevate western US surface ozone to unhealthy levels during spring. These intrusions can be classified as ‘exceptional events', which are not counted towards non-attainment determinations. Understanding the factors driving the year-to-year variability of these intrusions is thus relevant for effective implementation of the US ozone air quality standard. Here we use observations and model simulations to link these events to modes of climate variability. We show more frequent late spring stratospheric intrusions when the polar jet meanders towards the western United States, such as occurs following strong La Niña winters (Niño3.4<−1.0 °C). While El Niño leads to enhancements of upper tropospheric ozone, we find this influence does not reach surface air. Fewer and weaker intrusion events follow in the two springs after the 1991 volcanic eruption of Mt. Pinatubo. The linkage between La Niña and western US stratospheric intrusions can be exploited to provide a few months of lead time during which preparations could be made to deploy targeted measurements aimed at identifying these exceptional events.

## Abstract (web summary)

Deep stratospheric ozone intrusions can elevate western US ground-level ozone to unhealthy concentrations, but the factors driving interannual variability are poorly understood. Here, the authors combine observations and numerical simulations showing a link between intrusion events and strong La Niña winters.

## Graphical abstract

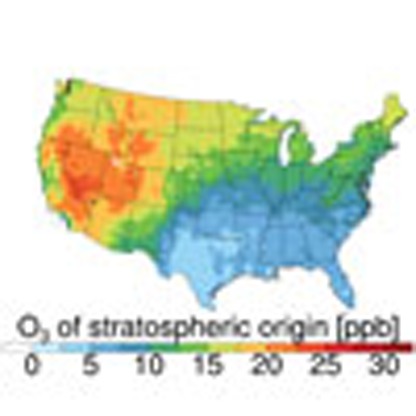

High concentrations of ground-level ozone adversely affect human health and ecosystem productivity^1^. To lessen these impacts, the US Environmental Protection Agency is proposing to lower the National Ambient Air Quality Standard (NAAQS) for ozone from 75 to 65–70 p.p.b.v. (ref. 2). Increasing mean baseline ozone from rising Asian emissions^3^,^4^,^5^,^6^,^7^, more frequent wildfires in summer^8^,^9^,^10^, and poorly understood deep stratospheric intrusions during spring^11^,^12^,^13^ may pose challenges in attaining more stringent ozone standards at high-elevation western US (WUS) regions. A recent field campaign identified 13 stratospheric intrusion events elevating WUS surface ozone during April–June 2010, and suggested that they may occur with sufficient frequency as to confound NAAQS attainment, particularly under a lower level of the standard^12^. While the Clean Air Act allows for screening of such ‘exceptional events' from counting towards non-attainment determinations^14^, failure to identify them accurately would imply a need for additional controls on regional emissions from human activities in order to attain the NAAQS. How the frequency of stratospheric intrusions varies from year to year and what controls its variability has not been examined, but is directly relevant to an effective implementation of the ozone NAAQS in WUS states.

The El Southern Oscillation (ENSO) is a dominant mode of global climate variability on interannual time scales. Prior work has identified ∼20–30% increases in mean upper tropospheric ozone abundances (∼2% in the middle troposphere) at northern mid-latitudes in winter and spring a few months after the peak of an El Niño event, which is attributed to increased stratosphere-to-troposphere transport (STT) of ozone^15^,^16^,^17^,^18^ (Supplementary Table 1 and Supplementary Note 1). The time lag is consistent with a response to changes in the stratospheric circulation that increase the ozone burden in the extratropical lower stratosphere^18^,^19^,^20^. The extent to which these upper tropospheric and lower-stratospheric (UTLS) ozone enhancements reach the surface is poorly characterized. A few studies have noted strong correlations between annual mean ozone in the lower stratosphere and in the lower troposphere, particularly over Europe and Canada^21^,^22^,^23^,^24^. In late spring, the cross-tropopause mass flux in the northern hemisphere peaks seasonally^25^, and the planetary boundary layer mixing is deeper than in winter. No previous studies have examined the role of STT on interannual variability of springtime high-ozone episodes measured in surface air that has direct implications for air quality and human health. Over the high-elevation WUS, deep STT of ozone into the boundary layer during spring has been shown to be a factor of 4 greater than at other northern mid-latitude regions^26^,^27^.

Here we show that the increased frequency of deep tropopause folds that form in upper-level frontal zones following strong La Niña winters exerts a stronger influence on springtime ozone levels at the WUS surface than the El Niño-related increase in UTLS ozone burden. We find that much of the year-to-year variability in springtime high-ozone episodes measured at high-elevation WUS sites is tied to known modes of climate variability, which modulate meandering in the polar frontal jet conducive to deep stratospheric ozone intrusions. A 34-year hindcast simulation with the Geophysical Fluid Dynamics Laboratory global chemistry–climate model (GFDL AM3; refs 12, 28, 29), nudged to reanalysis winds from 1979 to 2012 and with anthropogenic emissions held constant in time, enables us to relate observed ozone signals to changes in atmospheric circulation (see Methods section).

## Results

### Observed variability in WUS surface ozone

**Figure 1:**
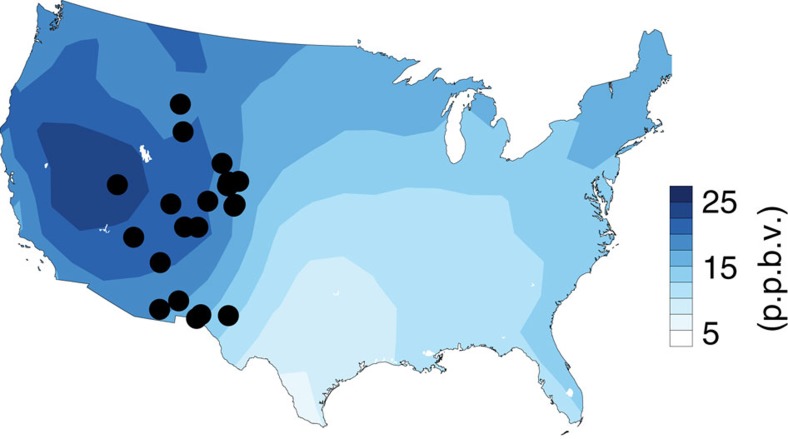
Mean stratospheric contribution to US surface ozone during April–May. The 23-year climatology of O_3_Strat from the model surface level is shown. Black filled circles denote locations of 22 surface ozone monitoring sites.

**Figure 2:**
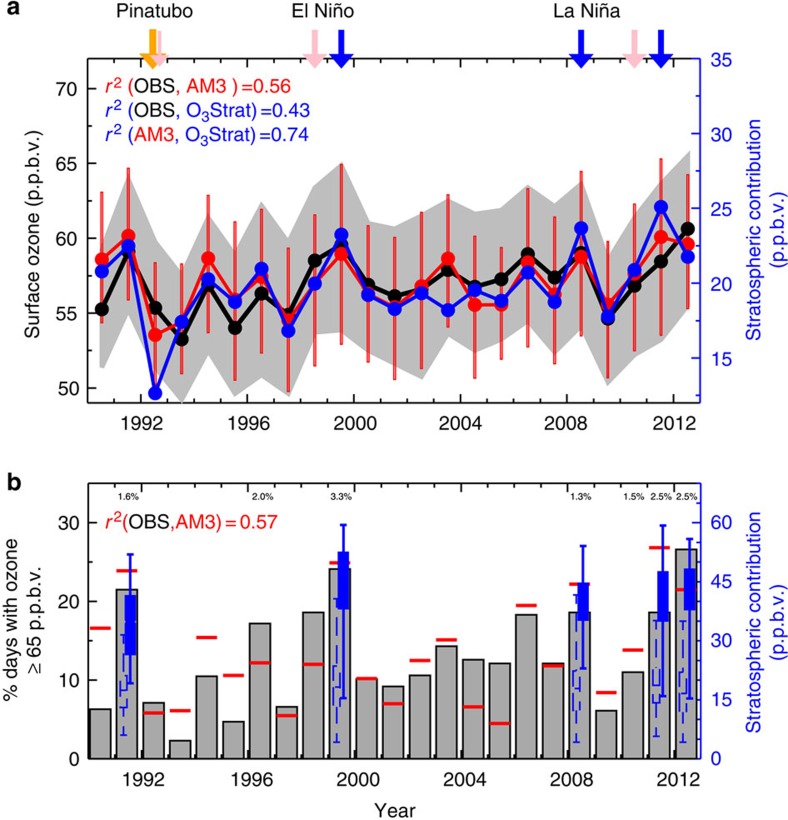
Interannual variability of springtime WUS surface ozone tied to stratospheric influence. (**a**) Median of daily MDA8 ozone at 22 high-elevation sites during April–May from 1990 to 2012 as observed (black) and simulated (red) by the GFDL AM3 model with fixed anthropogenic emissions; grey shading and red bars represent the 25th–75th percentiles. The median stratospheric influence for each year (O_3_Strat, blue) is shown using right axis. (**b**) Observed (grey) versus model (red) percentage of site-days with MDA8 ozone ≥65 p.p.b.v.; numbers along the top axis are the observed percentage of MDA8 ozone ≥75 p.p.b.v. (≤1% in other years; see Methods section). The blue box-and-whisker plots give the minimum, 25th–75th percentiles and maximum of stratospheric contribution (right axis) on days when total simulated ozone is below 60 p.p.b.v. (dashed) versus above 70 p.p.b.v. (filled) for the high-ozone springs. Arrows at the top of the graph indicate the springs following the Mt. Pinatubo volcanic eruption (orange), strong El Niño (pink) and La Niña (blue) winters (Fig. 6a).

We analyse hourly surface ozone measurements available from 22 high-elevation sites from 1990 to 2012 (Fig. 1 and Supplementary Table 2). We focus on the 1990–2012 period since continuous hourly ozone measurements are not available before then. Figure 2 shows year-to-year variability in mean daily maximum 8-h average (MDA8) ozone and days with MDA8 ozone above 65 p.p.b.v.—a possible future US ozone standard^2^—in surface air during April–May, when the frequency of stratospheric intrusions reaching the WUS surface peaks seasonally. Observations indicate that both mean ozone and the frequency of high-ozone events in WUS surface air decreased for the two springs following the eruption of the Mt. Pinatubo volcano in June 1991, but increased in the springs following strong La Niña winters in 1998–1999, 2007–2008 and 2010–2011. In contrast, little difference from neutral years is discernible for springs following strong El Niño events. For instance, 20 MDA8 measurement samples (3.3% of 602 site-days) exceeded the current 75 p.p.b.v. US standard in April–May following the 1998–1999 extreme La Niña event, compared with only seven samples (1% of 698 site-days) following the 1997–1998 extreme El Niño event and three samples (<0.6% of ∼500 site-days) in 1992–1993 following the Mt. Pinatubo eruption (Supplementary Table 3). Anomalously frequent high-ozone events were also observed during the late springs of 1991 and 2012, when the polar jet stream was unusually contorted over the WUS as discussed later in the paper.

**Figure 3:**
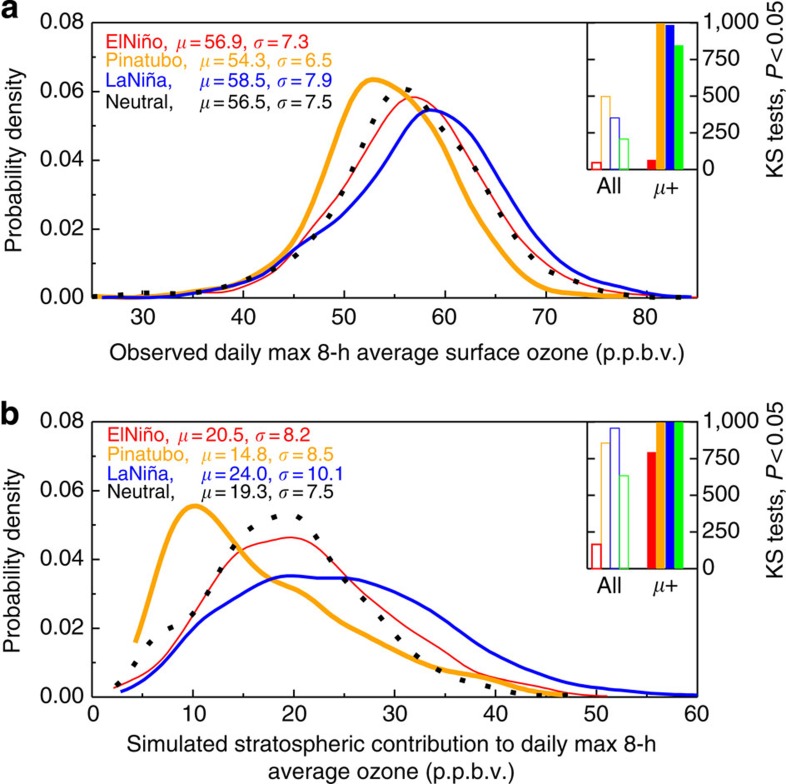
Shifts in the high tail of WUS surface ozone distributions tied to La Niña events and volcanic eruptions. (**a**,**b**) Probability distributions for observed MDA8 ozone (**a**) and simulated O_3_Strat (**b**) sampled in the model surface level during April–May following neutral (2004, 2005; dotted lines), El Niño (1998, 2010; red lines) and La Niña (1999, 2008, 2011; blue lines) conditions, and the Mt. Pinatubo eruption (1992, 1993; orange lines). The median (*μ*) and standard deviation (*σ*) are shown (p.p.b.v.). The inset shows the number of KS tests (1,000 total) where the difference in the entire distribution (open bars) and in the values above the median (filled bars), respectively, are statistically significant (at the *P*=0.05 level) for El Niño versus neutral (red), Mt. Pinatubo versus neutral (orange), La Niña versus neutral (blue) and La Niña versus El Niño (green) years.

Observed springtime variability in WUS surface ozone in association with La Niña events and with the Mt. Pinatubo eruption manifests as a statistically significant shift, upwards and downwards respectively, in the high tails of the MDA8 ozone probability distribution, in contrast to an ENSO-neutral spring (Fig. 3a). We investigate the similarities of surface ozone distributions associated with these different climate states with a Kolmogorov–Smirnov (KS) test, the nonparametric test for the equality of continuous one-dimensional distributions (see Methods section). The number of samples out of 1,000 KS tests for which a significant difference occurs (that is, the null hypothesis, that the distributions are drawn from the same population, is rejected; *P* value <0.05) is summarized in the top right corner of Fig. 3. Considering the entire distribution, we find ‘structural' but insignificant differences (open bars) between La Niña and El Niño. However, focusing the analysis on the high tails, that is, the right side of the distributions above the respective median values, we find significant differences (filled bars) for both La Niña and Mt. Pinatubo compared with neutral and El Niño springs.

Overall, the observations show a weaker day-to-day variability (s.d. *σ*=6.5 p.p.b.v.) following the Mt. Pinatubo eruption and a stronger day-to-day variability (*σ*=7.9 p.p.b.v.) following La Niña (Fig. 3a), consistent with a lower frequency of high-ozone events following Mt. Pinatubo and a higher frequency following La Niña (Fig. 2b). The difference in the surface ozone variances between Mt. Pinatubo and La Niña conditions is statistically significant (*P*<0.05) according to a Levene's test^30^. Despite the large El Niño enhancements to mean UTLS ozone burdens over northern mid-latitudes reported previously^15^,^16^,^17^,^18^,^19^,^20^, we find little overall change in observed WUS surface ozone in the springs following the strong El Niño events of 1997–1998 and 2009–2010. Our analysis below reconciles these findings by contrasting interannual variability in mean ozone aloft versus in the frequency of stratospheric intrusions that enhance WUS surface ozone.

### Stratospheric impacts on high-ozone events in surface air

We quantify stratospheric influence with a stratospheric ozone tracer (O_3_Strat) defined in the GFDL AM3 model relative to a dynamically varying tropopause, accounting for tropospheric loss^12^ (see Methods section). GFDL AM3 is one of a few models with a well-resolved stratosphere and comprehensive tropospheric chemistry, both of which are required to assess confidently stratospheric influence on tropospheric ozone. Analysis of daily ozonesondes, lidar and satellite observations indicates that AM3 captures the salient features of deep stratospheric intrusions and their impacts on day-to-day and vertical variations of WUS ozone^12^,^31^ (see Model evaluation in Methods section and Supplementary Figs 1–4).

The GFDL AM3 model with anthropogenic emissions held constant captures much of the observed interannual variability in median ozone (*r*^*2*^=0.56; Fig. 2a) and in the frequency of high-ozone events in WUS surface air (*r*^*2*^=0.57; Fig. 2b), lending confidence in its ability to relate the observed variability to specific processes. The model attributes much of the observed interannual variability to fluctuations in the stratospheric influence, with O_3_Strat fluctuations explaining 43% of the observed and 74% of the total simulated variability. In contrast, the role of Asian pollution^7^ and wildfires on interannual variability during spring is minimal (Supplementary Fig. 5). Model deficiencies in representing the complex ozone chemistry in fire plumes^32^ are of minor importance in spring when wildfires are less frequent than summer.

Prior work shows that transport of stratospheric ozone to the WUS surface during spring is mainly associated with deep tropopause folds^11^,^12^,^13^,^27^ that contribute to the highest observed surface ozone events^12^. Such tropopause folds occurred frequently during the springs of 1991, 1999, 2008, 2011 and 2012. During these years, the median value of the stratospheric contribution increased by as much as 30 p.p.b.v. on days when total surface ozone was above 70 p.p.b.v. versus below 60 p.p.b.v. in the model (filled versus dashed blue bars in Fig. 2b). The high-altitude WUS differs from low-elevation eastern US regions where the stratospheric contribution is typically below 10 p.p.b.v. in spring (Fig. 1) and is even lower during summertime pollution events that are dominated by ozone produced from US anthropogenic emissions^12^,^33^,^34^.

A significant increase in daily surface O_3_Strat above the median value occurs for La Niña compared with El Niño or neutral springs in the model (Fig. 3b), corresponding to the observed ozone shift (Fig. 3a). Following the Mt. Pinatubo eruption, however, O_3_Strat above the median declines substantially. For the entire distribution of daily O_3_Strat, significant differences occur for both La Niña and Mt. Pinatubo compared with neutral springs, but not for El Niño, paralleling the shifts in the overall observed daily ozone distribution. The model captures the observed shifts in the distribution of daily surface ozone associated with ENSO and with volcanic impacts (Supplementary Fig. 6).

**Figure 4:**
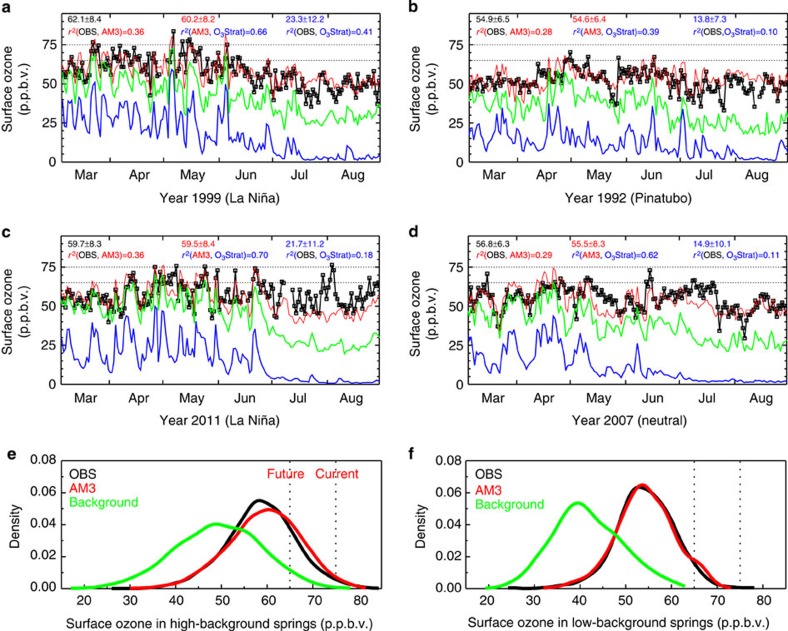
More frequent stratospheric intrusions elevating WUS surface ozone during La Niña springs. (**a**–**d**) Stratospheric and background contributions to surface MDA8 ozone for March through August at Gothic, Colorado in 1999 (**a**) versus 1992 (**b**) and at Chiricahua, Arizona in 2011 (**c**) versus 2007 (**d**). (**e**,**f**) Probability density functions at 22 sites during April–May for La Niña plus the high-ozone springs of 1991 and 2012 (**e**) and for the low-ozone springs of 1992–1993 (**f**). Shown are observations (black) and model simulations for total ozone (red), background ozone (green; estimated by shutting off North American anthropogenic emissions, see Methods), and the stratospheric contribution (blue). Dashed lines denote current (75 p.p.b.v.) and proposed (65 p.p.b.v.) US ozone standards. Statistics in **(a**–**d)** are for April–June.

Time series analysis of daily MDA8 ozone further supports a higher frequency of high-ozone events in WUS surface air following La Niña and during other high-ozone springs as a result of deep stratospheric intrusions (Fig. 4 and Supplementary Figs 7–10). For example, observed MDA8 ozone at Gothic in the Colorado Rocky Mountains (2,926 m altitude) shows a greater day-to-day variability and a higher mean concentration during April–June in 1999 (62.1±8.4 p.p.b.v.) than in 1992 (54.9±6.5 p.p.b.v.). Correlating model O_3_Strat with observations indicates that much of the variability in 1999 can be explained by fluctuations in stratospheric influence (*r*^*2*^=0.41; 23.3±12.0 p.p.b.v.). In contrast, fewer high-ozone events and weaker correlation with O_3_Strat occurred in 1992 (*r*^*2*^=0.10; 13.8±7.3 p.p.b.v.). The springtime MDA8 ozone in 1999 and 2011, both preceded by a strong La Niña winter, fluctuates strongly on shorter timescales than in 1992 or 2007 (Fig. 4a,c versus b,d), consistent with the transient, localized nature of deep tropopause folding events. Supporting this conclusion, the autocorrelation timescales for springtime MDA8 ozone following the 1998–1999 and 2010–2011 La Niña events are about half of those in 1992 and 2007 (Supplementary Fig. 11). We discuss additional time series analysis in Supplementary Note 2.

These deep intrusion events can push observed surface ozone levels to approach or exceed the 75-p.p.b.v. NAAQS level. If such exceptional events were not properly screened, they could affect attainment status, with more frequent impact for lower levels of the standard (for example, 65 p.p.b.v.). Even in the absence of North American anthropogenic emissions, daily MDA8 background ozone at WUS sites would exceed 60 p.p.b.v. on 13% of site-days in the model during the high-ozone springs when deep stratospheric intrusions were particularly active (Fig.4e versus f). During these seasons with higher background, there is less room for the addition of domestically produced ozone to achieve a targeted level of air quality.

### Changes in mean ozone aloft

**Figure 5:**
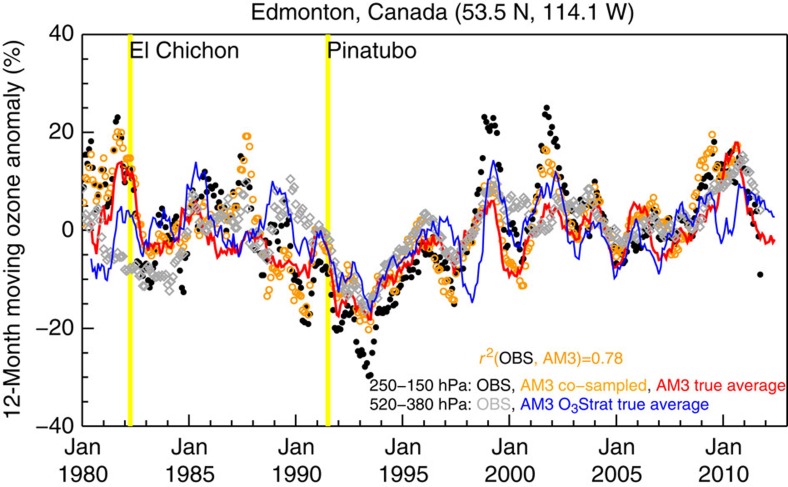
Covariance in mean ozone aloft in association with the Mt. Pinatubo eruption. 12-Month running mean anomalies (relative to the record mean) of lower-stratospheric to mid-tropospheric ozone above Edmonton in Southern Canada for 1980–2012. Symbols denote the averages of available weekly samples: from ozonesondes at 250–150 hPa (black circles) and 520–380 hPa (grey diamonds), and from the model at 250–150 hPa (orange circles). Solid lines show the model ‘true averages' of continuous daily sampling for ozone at 250–150 hPa (red) and tracer of stratospheric ozone at 520–380 hPa (blue).

**Figure 6 f6:**
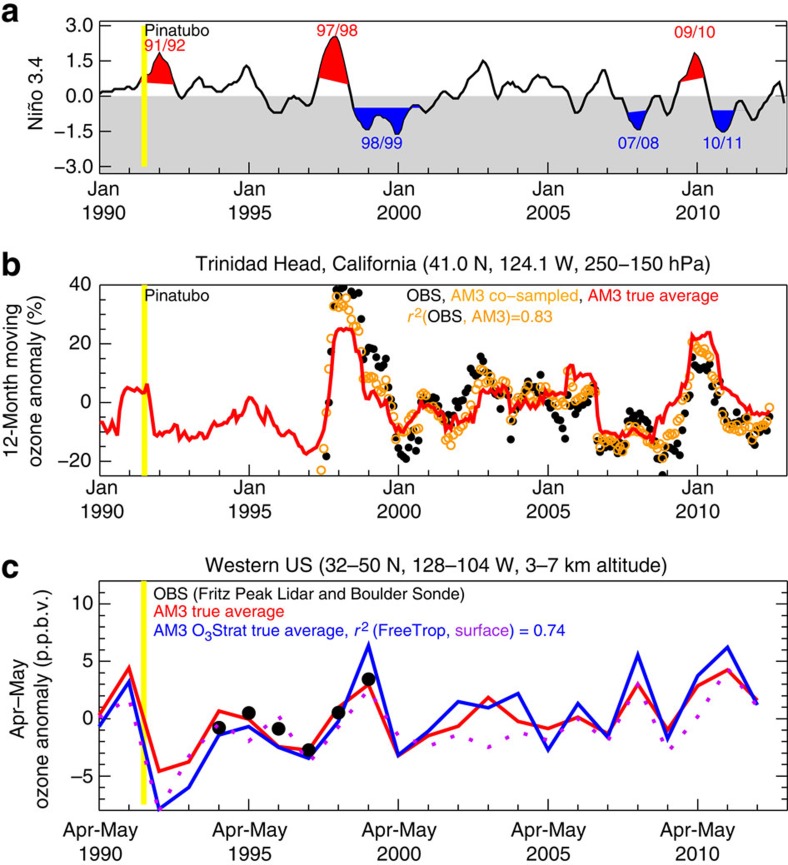
WUS surface ozone variability correlates strongly with ozone in the free troposphere but poorly with that in the UTLS. (**a**) The 1990–2012 Niño 3.4 index, highlighting strong El Niño (red) and La Niña (blue) events (see Methods section). (**b**) 12-Month running mean anomalies (relative to the record mean) of 250–150 hPa ozone at Trinidad Head for 1990–2012: from ozonesondes with weekly sampling (black circles), from the model co-sampled on sonde launch days (orange circles) and from the model ‘true average' of continuous daily sampling (red lines). (**c**) Mean WUS mid-tropospheric ozone anomaly in April–May: from the combined record of Fritz Peak lidar and Boulder sonde in Colorado (black circles; see Methods section) and from the model true averages of ozone (red) and tracer of stratospheric ozone (blue) over the entire domain (box in Fig. 7a). Also shown are anomalies in mean stratospheric contribution to surface ozone (dashed purple). Continuous ozonesonde profile measurements are not available at WUS sites in the 1980s. The mid-tropospheric ozonesonde records at Trinidad Head and Boulder are not shown in **c** because the available weekly sonde measurements were too infrequent to capture the actual variability (see Methods section).

We next examine the extent to which WUS surface ozone variability correlates with mean ozone levels in the free troposphere and in the lower stratosphere. Continuous ozone profile measurements are limited. We use the 12-month running average of weekly ozonesonde profile measurements averaged over 250–150 hPa to explore large-scale variability of UTLS ozone over high-latitude (Edmonton; Fig. 5) versus mid-latitude (Trinidad Head; Fig. 6a,b) regions in western North America. For WUS free tropospheric ozone, we focus our analysis in April–May when the strong influence from STT is most likely to reach surface air due to deep mixing depths (Fig. 6c). Variability of WUS free tropospheric ozone in summer and fall may reflect processes other than STT. To determine robust changes in the time evolution of ozone aloft, we compare observational records and model results co-sampled with observations in space and time with the ‘true average' (that is, continuous temporal sampling) determined from daily ozone fields archived from the model. Our analysis indicates that the available weekly ozone profile measurements at Trinidad Head and Boulder were too infrequent to capture the actual variability of mean mid-tropospheric ozone in April–May (see Methods section). For 12-month running averages, the weekly sampling is sufficient to capture most of the variability (Figs 5 and 6b).

The Edmonton sonde records show ∼20–30% declines of annual mean ozone at 250–150 hPa following the Mt. El Chichón (1982) and Mt. Pinatubo (1991) eruptions (Fig. 5). These declines have been attributed to loss of ozone resulting from heterogeneous chemistry on volcanic aerosols in the presence of anthropogenic chlorine^35^,^36^. Overall, annual mean ozone above Edmonton in the free troposphere correlates with that in the lower stratosphere (grey versus black in Fig. 5; *r*^*2*^=0.37): both reach record low levels in 1992–1993 and rebound afterwards as at other high-latitude (>50° N) sonde sites^22^,^37^. This similarity between the ozone concentrations in the UTLS and in the free troposphere above Edmonton probably reflects the much greater frequency of shallow tropopause folds over high-latitude regions^26^,^27^.

In contrast, the stratospheric influence over the mid-latitude WUS is driven mainly by less frequent, but much deeper tropopause folds, thereby decreasing the correlation between ozone in the UTLS and free troposphere (Fig. 6b versus c). Observed mean UTLS ozone over Trinidad Head increased by as much as 20–40% in late winter to spring following the strong El Niño events of 1997–1998 and 2009–2010 (Fig. 6a,b). Satellite observations show ∼25% enhancements in total ozone columns over the Northwest United States in May 1998 and 2010 (Supplementary Fig. 12). Consistent with the limited available observations, AM3 indicates 20–40% differences in mean UTLS ozone burdens over the WUS for all moderate-to-strong El Niño (four total) versus La Niña (four total) events during the past 34 years, except for the 1991–1992 El Niño following the Mt. Pinatubo eruption. These ozone increases are most pronounced 2–4 months after tropical Pacific warming peaks, consistent with the known delayed response of northern extratropical UTLS ozone to El Niño^15^,^16^,^17^,^18^. Recent studies suggest that El Niño and the easterly shear Quasi-Biennial Oscillation act to strengthen the stratospheric overturning circulation and hence increase transport of stratospheric air polewards and downwards to the mid-latitude upper troposphere^18^,^19^,^20^. AM3 captures the observed ozone increases that are signatures of this stratosphere-troposphere coupling: the simulated and observed UTLS ozone interannual variability at Trinidad Head correlate strongly (*r*^*2*^=∼0.8). The simulated stratospheric ozone signal associated with the 1991–1992 El Niño is masked by the Mt. Pinatubo eruption consistent with prior analysis of satellite observations^20^.

Despite lower ozone levels in the UTLS during La Niña than El Niño (Fig. 6a,b), the AM3 ‘true average' shows that late spring mid-tropospheric ozone and O_3_Strat over the WUS are highest following the three La Niña winters of 1998–1999, 2007–2008 and 2010–2011 (Fig. 6c). Langford^15^ noted positive correlations between mid-tropospheric and lower-stratospheric ozone observed at Fritz Peak, Colorado during 1994–1998 (without La Niña years), reflecting higher than neutral ozone levels during the El Niño events of 1994–1995 (weak) and 1997–1998 (strong). For this short record, AM3 captures the observed relationship (*r*^*2*^=0.69) but when the entire 1990–2012 period (including both El Niño and La Niña years) is considered, AM3 indicates little correlation (*r*^*2*^=0.18) between mid-tropospheric and lower-stratospheric ozone over the WUS. An extension of the Fritz Peak record to 1999 shows that, as indicated by the model, the mid-tropospheric ozone anomaly in April–May is higher following the La Niña winter of 1998–1999 than in either El Niño or neutral conditions (black circles in Fig. 6c). We conclude that WUS surface ozone variability correlates poorly with ozone burdens in the UTLS (*r*^*2*^=0.07; Fig. 2a versus 6b, [Fig f6]) but strongly with that in the free troposphere (*r*^*2*^=0.74; Fig. 2a versus 6c, [Fig f6]).

The ENSO cycle has favoured the La Niña phase since the extreme El Niño event of 1997–98 (ref. 38), which our findings indicate would lead to a stronger stratospheric influence on WUS ozone but weaker transport of Asian pollution towards the eastern Pacific^29^ and the WUS in recent decades relative to prior decades (Supplementary Fig. 5). Significant year-to-year variability in stratospheric influence can complicate the unambiguous attribution of observed ozone trends in WUS free tropospheric and surface air, particularly in short records. Prior work has implicated the role of rising Asian emissions during the 1990s and the 2000s in driving springtime ozone increases observed at some WUS sites^6^,^39^,^40^,^41^. We speculate that fluctuations in stratospheric influence contribute to the overall upward ozone changes at high-elevation WUS sites during 1992–1999 and 2004–2012 (Fig. 2a). In particular, stratospheric influence increases during 1992–1999 both in the free troposphere (1.44±0.99 p.p.b.v. per year) and in surface air (0.94±0.91 p.p.b.v. per year) in the model coincide with the observed surface ozone increase (0.82±0.79 p.p.b.v. per year). The simulated increasing stratospheric influence during the 1990s has also been documented in other northern mid-latitude regions^21^,^23^.

### Synoptic variability tied to climate regimes

**Figure 7:**
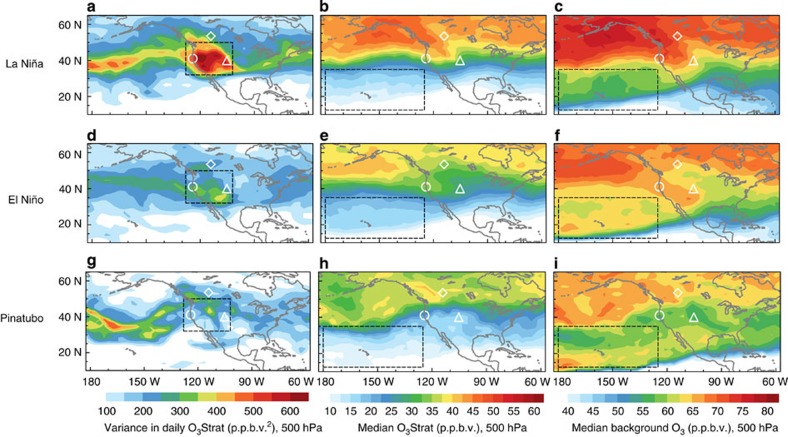
Strong day-to-day fluctuations of WUS free tropospheric ozone driven by meandering jet and tropopause folds during La Niña springs. (**a**–**c**) Simulated variance in daily O_3_Strat (**a**), median of daily O_3_Strat (**b**) and median of daily background ozone (**c**) at 500 hPa in April–May for the La Niña composite (1989, 1999, 2008 and 2011). (**d**–**f**) Same as **a**–**c** but for the El Niño composite (1983, 1987, 1998 and 2010). (**g**–**i**) Same as **a**–**c** but for 1992 following the Mt. Pinatubo volcanic eruption. Locations of Edmonton (◊), Trinidad Head (⊙) and Fritz Peak-Boulder sonde sites (▵) are shown. The boxes indicate the regions where ozone variability corresponds with jet characteristics for La Niña and El Niño (Fig. 8).

**Figure 8:**
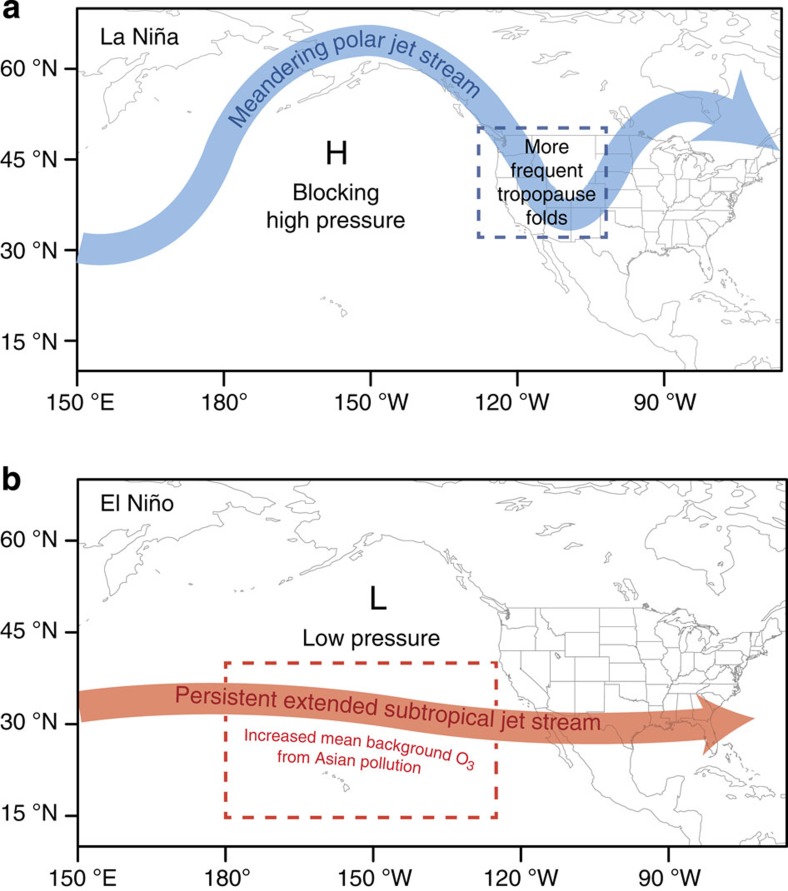
Schematic for mid-latitude jet characteristics and sources of lower tropospheric ozone variability in winter extending into the spring months during strong La Niña versus El Niño events. The blue box in **a** denotes where frequent deep tropopause folds occur as a result of the meandering polar jet over the central WUS associated with La Niña. The red box in **b** indicates where mean background ozone increases due to more pollution transport from Asia as a result of the equatorward shift and eastward extension of the subtropical jet associated with El Niño. The figure is adapted from ref. 47.

We next interpret modes of climate variability and associated meteorological mechanisms responsible for changes in mean versus synoptic variability of free tropospheric and surface ozone in the Pacific–North America sector (Figs 7 and 8). Prior analysis of daily ozonesondes, water vapour and lidar measurements indicates that the AM3 O_3_Strat tracer represents the episodic, layered structure of ozone enhancements in the free troposphere consistent with the observed characteristics of deep tropopause folds^12^. Here we compare the variance in daily O_3_Strat at 500 hPa during April–May to infer year-to-year variability in the frequency and intensity of deep tropopause folds (Fig. 7a,d,g). To place the short ozone observational records into a longer-term context, we increase the sample size (and statistical power) of strong El Niño and La Niña events by extending the analysis period to 1979–2012 for simulated ozone.

The primary mechanism for the transport of stratospheric ozone to the middle and lower troposphere is descent through the dry airstream of mid-latitude cyclones^42^,^43^. The wintertime responses of the mid-latitude storm track and regional climate over the North Pacific and North America to ENSO events are well known: Storms follow a more southern route into the southern US–Gulf of Mexico during El Niño and a more northern route into the Pacific Northwest during La Niña^44^,^45^,^46^,^47^ (Supplementary Fig. 13 and Supplementary Note 3). The responses in spring are less certain. We examine the spatial pattern of ozone variability in April–May following strong ENSO events (above ±1.0 °C anomaly), and find similarities to the known adjustments of the wintertime atmospheric flow across the eastern North Pacific and North America in response to tropical Pacific sea surface temperature (SST) anomalies (Figs 7 and 8).

The main characteristic of the flow pattern for La Niña is the presence of a large area of high pressure across the North Pacific and a wave-like jet stream over the United States of America and Canada^44^,^45^,^46^,^47^(Fig. 8a). In contrast to El Niño, the propagation of mid-latitude cyclones over the North Pacific shows a distinct northward shift towards the US Pacific Northwest region during La Niña^44^,^45^,^46^. These shifts are most prominent in winter but still persist in late spring for those La Niña episodes with strong SST cooling (>1.0 °C) in the tropical Pacific from December through March (Supplementary Fig. 14). The enhanced storm-track activity across the central WUS and associated meandering jet stream related to La Niña increase the frequency of cold frontal passages that facilitate deep tropopause folds and isentropic transport of stratospheric ozone into the lower troposphere. Despite the weakened storm-track activity in April–May compared with January–February, the greater cross-tropopause ozone flux in late spring^25^ amplifies the signals of ENSO in ozone. Supporting this conclusion, the springtime variance in daily O_3_Strat at 500 hPa over the WUS is a factor of two greater following La Niña than El Niño (Fig. 7a versus d). The greater ozone variability occurs in each La Niña versus El Niño event during 1979–2012 (Supplementary Fig. 15). Stronger day-to-day fluctuations in stratospheric influence at 500 hPa during La Niña springs are consistent with the significant shifts in the upper half of the daily ozone and O_3_Strat distributions at surface sites (Fig. 3). In contrast, the influence of STT on surface ozone during winter is limited, since most stratospheric intrusions during winter do not reach surface air due to boundary layer inversions.

During El Niño, the most prominent change in the atmospheric circulation over the Pacific–North America sector is an eastward extension and equatorward shift of the subtropical jet stream and storm track from the International Date Line to the southern US–Gulf of Mexico^44^,^46^,^47^ (Fig. 8b). These shifts enhance long-range transport of Asian pollution in late winter and spring following the peak of an El Niño^17^,^29^, increasing free tropospheric background ozone over the eastern North Pacific relative to La Niña (Fig. 7f versus c). In contrast to the polar frontal jet, tropopause folds at the subtropical jet do not penetrate as deeply into the troposphere, as the frontal zone is less steeply sloped^48^. Thus, the dominant change (relative to neutral) in the stratospheric influence during El Niño occurs via a mean contribution to the mid-troposphere (Figs 6c and 7e; and ref. 15). This interpretation is consistent with the El Niño versus La Niña composite analysis of probability distributions of springtime daily ozone measured at Mauna Loa (3.4 km altitude), Hawaii over the past 40 years^29^.

Both observations and climate models show that strong equatorial volcanic eruptions have been followed by a pronounced positive phase of the Arctic Oscillation (AO) for one or two boreal winters^36^,^49^. The positive AO is characterized by the polar jet blowing strongly and consistently from west to east, as it did in 1992 following the Mt. Pinatubo eruption (Supplementary Fig. 16a), keeping ozone-rich cold Arctic air locked in the polar region^50^. A weak variance in simulated daily O_3_Strat at 500 hPa and a lower mean concentration occurred over the WUS in spring 1992, reflecting fewer southward intrusions of upper Arctic air (Fig. 7g–i). Previous work suggests a decrease in the global annual mean STT of ozone in 1992–1993 (ref. 51). Here we find that the Mt. Pinatubo eruption also modulated WUS surface ozone, with a significant reduction in high-ozone events due to fewer tropopause folds accompanying the positive AO.

The mid-latitude atmosphere shows a high level of internal variability in addition to the forced response to tropical ocean temperatures and to volcanic aerosols. The polar jet stream was unusually contorted during the late springs of 1991 and 2012, facilitating mixing of ozone-rich, stratospheric air into the WUS lower troposphere (Supplementary Fig. 16b,c). The meandering jet stream, conducive to deep tropopause folds, contributed to the anomalously frequent high-ozone events observed at the WUS surface in 1991 and 2012 (Fig. 2). Given a high level of internal variability, the mid-latitude atmospheric responses differ during individual ENSO events^44^. Simulated mean stratospheric contribution to springtime WUS surface ozone following the La Niña winter of 1988–1989 is not as prominent as those La Niña events in recent decades (Supplementary Fig. 17). Nevertheless, simulated enhancements of stratospheric contribution during the 1990s–2000s La Niña events are noticeable from all El Niño and neutral years (except 1991) over the entire 1980–2012 period. Future work should examine the role of internal variability in addition to La Niña in contributing to stratospheric intrusion frequency and surface ozone variability over the WUS. The relationship between the polar jet and the frequency of surface high-ozone events documented here serves as motivation, in addition to the relationships with weather extremes, to develop a mechanistic understanding of the dynamical processes driving variability in the location and meandering of the mid-latitude jet^52^,^53^,^54^.

## Discussion

We conclude that the frequency of springtime high-ozone episodes observed at high-elevation WUS sites is linked to interannual variability in the meandering of the polar jet such as occurs during the ENSO cycle and following large volcanic eruptions. Such modes of climate variability modulate WUS ozone air quality by changing the frequency of deep stratospheric ozone intrusions. These intrusion events occur most frequently when the polar jet stream meanders towards the central WUS (Fig. 8a) as it does following some strong La Niña episodes in the tropical Pacific. Despite well-documented enhancements in mid-latitude UTLS ozone following El Niño^15^,^16^,^17^,^18^,^19^,^20^, we find that these ozone enhancements do not reach WUS surface air. We underscore that ozone produced from US anthropogenic emissions dominates pollution events during summer and at low-elevation US regions. Nevertheless, stratospheric intrusions reaching surface air can occur with sufficient frequency in spring when the polar jet stream is unusually contorted as to confound NAAQS attainment in high-elevation WUS regions.

Under a more stringent US national air quality standard for ground-level ozone^2^, stratospheric intrusion events would affect attainment status more frequently if they were not identified as ‘exceptional events' (Fig. 4). A working group consisting of cross-agency air quality managers and scientists has been recently established to develop tools to forecast such events, identify days for ozone public health advisories^55^ and prepare ‘exceptional event' demonstrations^56^. Most El Niño and La Niña episodes develop in late spring to summer and peak near the end of the calendar year^44^. Our finding that deep stratospheric intrusions reaching WUS surface air occur more frequently in the spring following the mature winter phase of a strong La Niña episode raises the possibility of developing seasonal predictions with several months of lead time. Knowledge of the possibility of an upcoming active stratospheric intrusion spring season could allow WUS air quality planners to prepare accordingly, such as conducting daily forecasts for public health alerts and deploying targeted measurements aimed at identifying exceptional events. These targeted measurements can include additional ozonesonde or lidar profiles as well as high-frequency collocated measurements of surface ozone, carbon monoxide and water vapour mixing ratios at a number of high-elevation sites.

## Methods

### Surface measurements

Surface ozone data were obtained from 22 high-elevation (1.2–3.5 km altitude) rural monitoring sites, including Niwot Ridge Observatory operated by the US National Oceanic and Atmospheric Administration (NOAA), 13 sites in the US Clean Air Status and Trends Network (CASTNET) and 8 sites in the EPA Air Quality System (Supplementary Table 2). All analyses use the daily MDA8 value for each day and each monitor, which is the relevant policy metric (compliance with the ozone standard is based on the 3-year average of the annual fourth highest MDA8 value^1^,^2^). The number of days above 65 and 75 p.p.b.v. is calculated for each monitor and then summed across all sites in each April–May. Supplementary Table 3 summarizes statistics of MDA8 samples (number of sites multiplied by number of days with available data) for each April–May from 1990 through 2012. The sample size for each year ranges from 349 to 1,200 site-days, with more data available in recent years. Approximately 1–3% of MDA8 samples exceed the current NAAQS level of 75 p.p.b.v. during the high-ozone springs.

### Ozone profile measurements

Continuous ozone profile measurements are limited. We gather ozone profile measurements over western North America, which are available from (1) weekly ozonesondes at Edmonton, Canada since 1980, Trinidad Head, California since 1997 and Boulder, Colorado since 1993; (2) an ozone lidar at Fritz Peak near Boulder operated several times per week between July 1993 and June 1999 (ref. 15); and (3) daily ozonesonde measurements available at six sites in California during May–June 2010 (ref. 57).

We use the 12-month running average of ozonesonde measurements averaged over 250–150 hPa (∼50 daily profiles; ∼1,600 samples per year) to explore large-scale variability of UTLS ozone over western North America. Using a 12-month running average is appropriate because the variability in the annual mean of UTLS ozone is dominated by the prominent variability in spring (Supplementary Fig. 3). For free tropospheric ozone, we focus our analysis in April–May when the strong influence from STT is most likely to reach surface air due to deep mixing depths. Ozonesonde profiles, however, are available on fewer than 15% of days during April–May. Our comparison of the model co-sampled with the available weekly sonde profiles versus the ‘true' monthly model average of continuous temporal sampling indicates that the mid-tropospheric ozonesonde records for April–May do not represent the actual interannual ozone variability in the WUS free troposphere (Supplementary Fig. 18 and Supplementary Note 4). The sampling frequency has also been shown to substantially influence the analysis of tropospheric ozone changes over Europe from ozonesonde observations^58^. We thus combine the Boulder sonde profiles with ozone lidar profiles at Fritz Peak. The merged record (black circles in Fig. 6c) includes at least 165 samples at 4–6 km altitude on 30–43% of days for each April–May during 1994–1999 (Supplementary Table 4).

### Chemistry–climate model experiments

We conduct a set of hindcast simulations with the GFDL AM3 chemistry–climate model nudged to the National Center for Environmental Prediction and National Center for Atmospheric Research reanalysis winds^59^ from 1979 to 2012 as previously described^29^. We avoid using the reanalysis data for before 1979 owing to the discontinuity of satellite data assimilated into the reanalysis. Our chemistry–climate model simulation represents one of the longest simulations to date with interactive stratospheric and tropospheric chemistry and aerosols^60^,^61^ driven by observed meteorology during the entire satellite era.

The model has a horizontal resolution of ∼200 × 200 km^2^ and includes 48 vertical layers, ranging in thickness from ∼70 m near the Earth's surface to 1–1.5 km near the tropopause and 2–3 km in much of the stratosphere. This vertical resolution is considerably higher than those of most previous models used for attribution studies (Supplementary Table 1). The influence from major volcanic eruptions is imposed through the specification of monthly time series of zonal mean, multi-wavelength aerosol extinction as a function of altitude and latitude based on satellite measurements^49^,^62^. The global net STT of ozone in GFDL AM3 is ∼535 Tg per year, within the 400–600 Tg per year range derived from trace gas correlations observed in the lower stratosphere^63^.

Three AM3 simulations are designed to isolate the response of WUS ozone to historical changes in meteorology, in wildfire emissions and in background ozone (Supplementary Table 5). In the FIXEMIS and IAVFIRE simulations, the methane lower boundary condition for chemistry and anthropogenic emissions of non-methane ozone precursors are held constant in time in order to examine the role of meteorology alone. In the background simulation, anthropogenic emissions are set to zero over North America but vary from year to year elsewhere (Supplementary Note 5). Background ozone thus includes the contribution of ozone transported from the stratosphere, pollution from foreign continents and ozone produced by global methane, lightning NO_*x*_, wildfires and biogenic emissions.

### Quantifying stratospheric ozone in surface air

The O_3_Strat is defined relative to a dynamically varying e90 tropopause^64^ and is subject to chemical losses in the same manner as odd oxygen (O_*x*_=O_3_+O+O(^1^D)+NO_2_+2 × NO_3_+3 × N_2_O_5_+HO_2_NO_2_+PANs) of tropospheric origin and deposition to the surface^12^. Diagnosis of stratospheric air (e90<85 p.p.b.v.) and calculation of O_3_Strat loss in the troposphere are conducted at each 30-min model time step. Analysis of trace gas correlations and potential vorticity indicates that variability in AM3 O_3_Strat represents episodic ozone enhancements in the free troposphere and at the surface consistent with the salient features of deep stratospheric intrusions^12^,^31^. Ozone produced from tropospheric precursors may affect mean ozone levels in the stratosphere over a longer term^65^,^66^,^67^, thus the mean baseline level of AM3 O_3_Strat likely represents an upper limit of stratospheric influence. All analysis in this study has used O_3_Strat in the FIXEMIS simulation, in which the effects from year-to-year changes in anthropogenic emissions of tropospheric ozone precursors (including methane) on ozone above the e90 tropopause have been eliminated. Simulated interannual variability of O_3_Strat in the troposphere mainly represents the influences from changes in dynamics, volcanic aerosols and halocarbons.

### Model evaluation

The stratospheric intrusions that penetrate deeper into the troposphere typically appear as fine-scale filamentary structures in satellite images and in vertical profile measurements of ozone and related tracers^68^. We use near-daily ozonesonde observations available at six sites during May–June 2010 to evaluate the ability of the GFDL AM3 model at ∼200 × 200 km^2^ resolution in representing deep stratospheric intrusions. We also compare the ∼200 × 200 km^2^ AM3 simulation with a ∼50 × 50 km^2^ AM3 simulation available for January to June 2010 (ref. 12) to assess the impact of model resolution. Supplementary Figs 1 and 2 show that the GFDL AM3 model at both ∼50 × 50 and ∼200 × 200 km^2^ horizontal resolutions captures the day-to-day variability of observed ozone in the free troposphere, with correlation coefficients ranging from 0.55 to 0.95. The ∼50 × 50 km^2^ model typically better reproduces the day-to-day variability in the upper troposphere (6–9 km), given its better ability to resolve the filamentary structure of tropopause folds (Supplementary Fig. 1). The ∼200 × 200 km^2^ model reproduces the large-scale view of deep stratospheric intrusions and their impacts on day-to-day variability of ozone below 3 km at lower-latitude sites. This finding suggests that AM3 multidecadal simulations at ∼200 × 200 km^2^ horizontal resolution are suitable for quantifying regional-scale interannual variability of stratospheric influence on ozone in the WUS lower troposphere.

The GFDL AM3 model at ∼200 × 200 km^2^ resolution also reproduces many salient features of observed ozone above western North America, including interannual variability in the lower stratosphere (*r*^*2*^=∼0.8; Figs 5 and 6) and its springtime maximum (Supplementary Fig. 3), as well as mean vertical profiles in the troposphere during spring (Supplementary Fig. 4). Realistic simulations of ozone variability in the UTLS indicate that nudging does not introduce abnormal transport of ozone from the stratosphere. Prior analysis shows that the model without nudging, driven by observed SSTs, tends to be biased high in the free troposphere during winter months and in March^60^. We find that the model nudged to reanalysis winds has relatively small biases during April–May when episodes of stratospheric influence on WUS surface ozone peak seasonally (Supplementary Fig. 4).

We use ozone concentrations sampled in the model surface level for comparison with observations from the WUS rural monitoring sites. The model captures the observed shifts in the probability distribution of daily MDA8 ozone at WUS surface sites in April–May related to strong ENSO events and to the influence of volcanic aerosols from the eruption of Mt. Pinatubo (Supplementary Fig. 6). During the high-ozone springs of 1999, 2011, 1991 and 2012, the model captures observed synoptic variability of surface ozone at WUS high-elevation sites (*r*^*2*^=0.3–0.5) and attributes it mainly to stratospheric influence (*r*^*2*^=0.5–0.8 between O_3_Stat and simulated total ozone; Supplementary Figs 7–10). Using lidar and ozonesonde observations, Langford *et al*.^11^ documented a striking example of a deep tropopause fold bringing ∼215 p.p.b.v. of ozone to within 1 km of the highest peaks in the Colorado Rocky Mountains on 6 May 1999. Daily MDA8 ozone concentrations greater or equal to the current NAAQS level of 75 p.p.b.v. were recorded at a few monitoring sites. We find that the GFDL AM3 model captures this deep intrusion event, and indicates that the stratospheric influence increases by ∼40 p.p.b.v. above the baseline level on 6 May 1999 when surface MDA8 ozone reached 84 p.p.b.v. at Gothic in the Colorado Rocky Mountains (Fig. 4a and Supplementary Fig. 7).

### Significance testing

The KS test is used to test the differences in the daily surface ozone distributions between La Niña (1999, 2008 and 2011), El Niño (1998 and 2010), neutral (2004–2005) and Mt. Pinatubo (1992–1993) conditions (Fig. 3). To have a comparable sample size for the different climate states, we define neutral conditions as the springs of 2004–2005, when both observed ozone and model O_3_Strat represent the average level and no unusual changes in the location and meandering of the polar jet occur. Significant shifts in the upper half of the observed ozone and model O_3_Strat distributions also occur for La Niña conditions when compared with the composite of 2000–2002 or 2006–2009 neutral springs. The null hypothesis is that the individual data sets stem from populations with identical probability distribution. As it is known that large sample size and the presence of ties (that is, identical values) affects the robustness of *P* value estimates in the KS test, we apply a random sampling approach to test for similarities of these surface ozone distributions. We draw 1,000 random samples of size *n*=100 (without replacement) from the individual surface ozone distributions, perform KS tests for each sample pair and establish the overall test statistic from the *P* values of the 1,000 KS tests. A similar approach is applied to perform Levene's test^30^ for equality of the surface ozone variances between La Niña and Mt. Pinatubo conditions.

### Definition of ENSO events

The effects of ENSO on the storm track over the eastern North Pacific and North America is noticeable, especially during strong events, but these effects are weak and ambiguous in years with weaker events^44^,^69^. Thus, we focus our analysis on strong episodes defined as the Niño 3.4 index at or above ±1.0 °C anomaly for the overlapping 3-month periods of Dec–Jan–Feb, Jan–Feb–Mar and Feb–Mar–Apr, as opposed to using the ±0.5° threshold defined by the NOAA Climate Prediction Center. By this definition, the 66 years of the National Center for Environmental Prediction reanalysis include the strong El Niño events of 1957–1958, 1965–1966, 1968–1969, 1982–1983, 1986–1987, 1991–1992, 1997–1998 and 2009–2010, and the strong La Niña events of 1970–1971, 1973–1974, 1988–1989, 1998–1999, 2007–2008 and 2010–2011.

## Author contributions

M.L., A.M.F. and L.W.H. conceived the overall concept. M.L. designed the research, conducted model experiments, analysed the data and wrote the manuscript. A.O.L., S.J.O. and D.T. provided ozonesonde and lidar measurements, and guided their interpretation. H.E.R. conducted the statistical tests for surface ozone distributions. All authors contributed to discussions and edited the manuscript.

## Additional information

**How to cite this article:** Lin, M. *et al*. Climate variability modulates western US ozone air quality in spring via deep stratospheric intrusions. *Nat. Commun*. 6:7105 doi: 10.1038/ncomms8105 (2015).

## Supplementary Material

Supplementary Information Supplementary Figures 1-18, Supplementary Tables 1-5, Supplementary Notes 1-5 and Supplementary References
