# Supplementary material for: Climate variability modulates western US ozone air quality in spring via deep stratospheric intrusions
Source: Nat Commun. 2015 May 12;6:7105. doi: 10.1038/ncomms8105 (PMC4432627; doi:10.1038/ncomms8105)
Supplement: Supplementary Information — Supplementary Figures 1-18, Supplementary Tables 1-5, Supplementary Notes 1-5 and Supplementary References [file ncomms8105-s1.pdf]

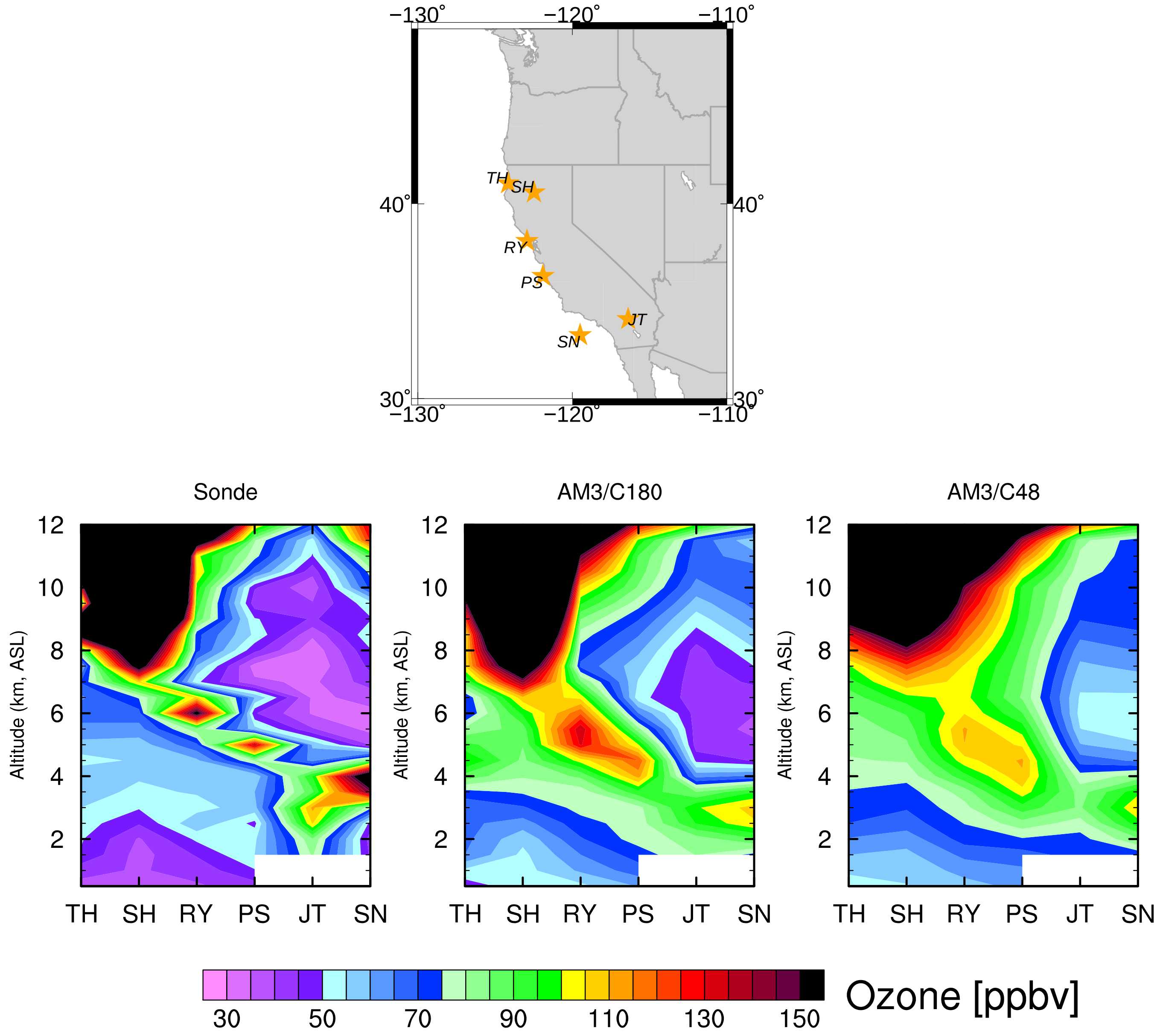

Supplementary Figure 1. **Evaluating model ability to represent deep stratospheric intrusions**. Curtain plots of ozone mixing ratios as a function of altitude at six sonde sites (orange stars on map) from north to south over California during a stratospheric intrusion event on 28 May 2010, as observed (left) and simulated by GFDL AM3 with ~50x50 km^2 (middle) and ~200x200 km^2 (right) horizontal resolutions.

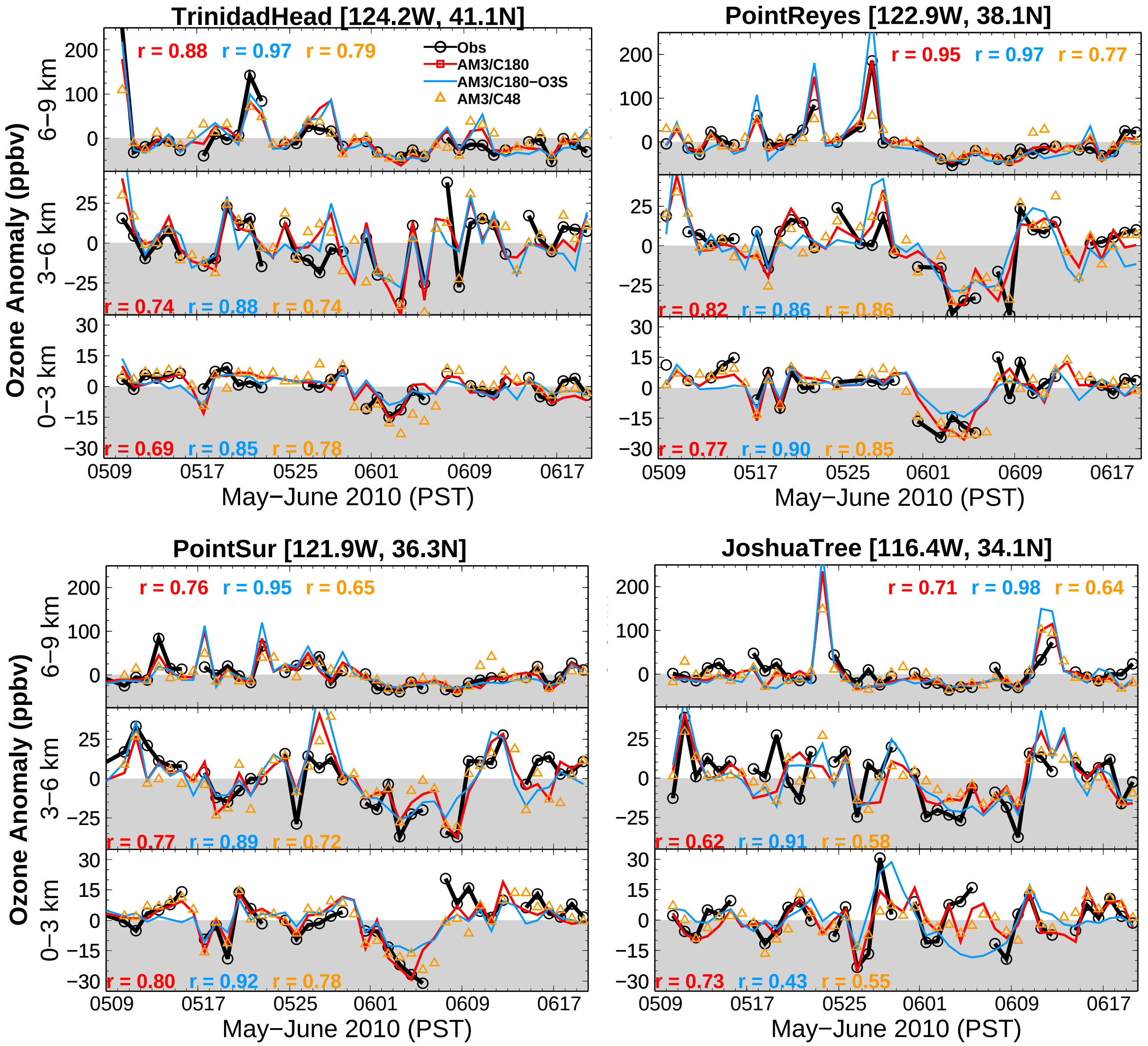

Supplementary Figure 2. Evaluating ozone vertical transport in the model with extensive campaign data during May-June 2010. Shown are daily ozone anomalies (relative to the record mean) averaged over 3-km altitude bins at Trinidad Head, Point Reyes, Point Sur, and Joshua Tree ozonesonde sites (see map in Supplementary Fig. 1) from May 9 through June 19 in 2010 as observed (black) and simulated by AM3 with ~50x50 km^2 (red lines) versus ~200x200 km^2 (orange triangles) horizontal resolution. A tracer of stratospheric ozone (O_3Strat, blue) and its correlation with simulated total ozone (r in blue) from the ~50x50 km^2 model are shown. Correlations between observed and simulated ozone are also shown.

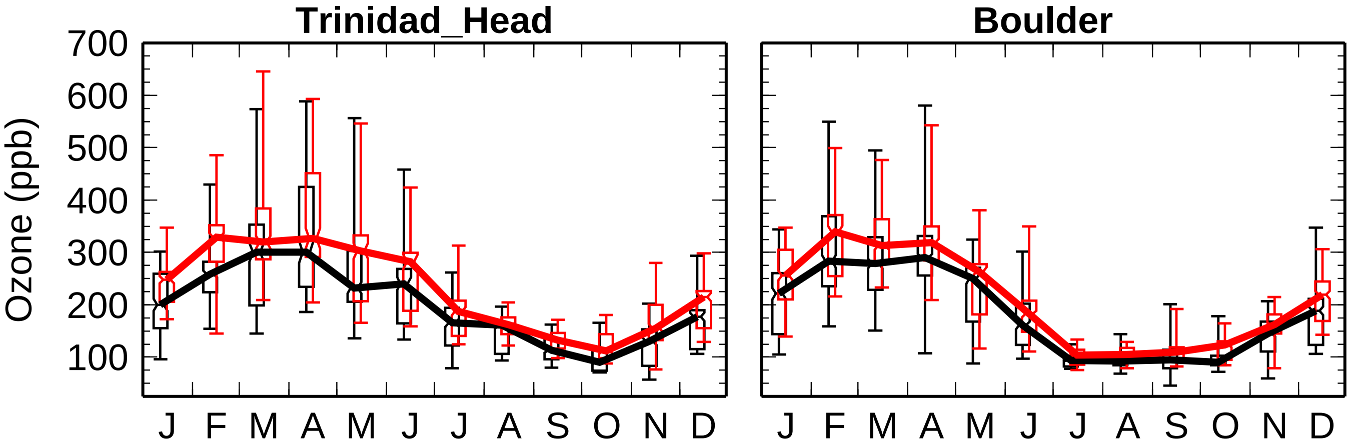

Supplementary Figure 3. **Seasonal cycle of 250-150 hPa ozone at Trinidad Head and Boulder sonde sites**. Observations (black) and simulations (red) by the GFDL AM3 model are shown. The box-and-whisker plots represent the min, 25th, 50th, 75th, max of monthly mean values during 1995-2012.

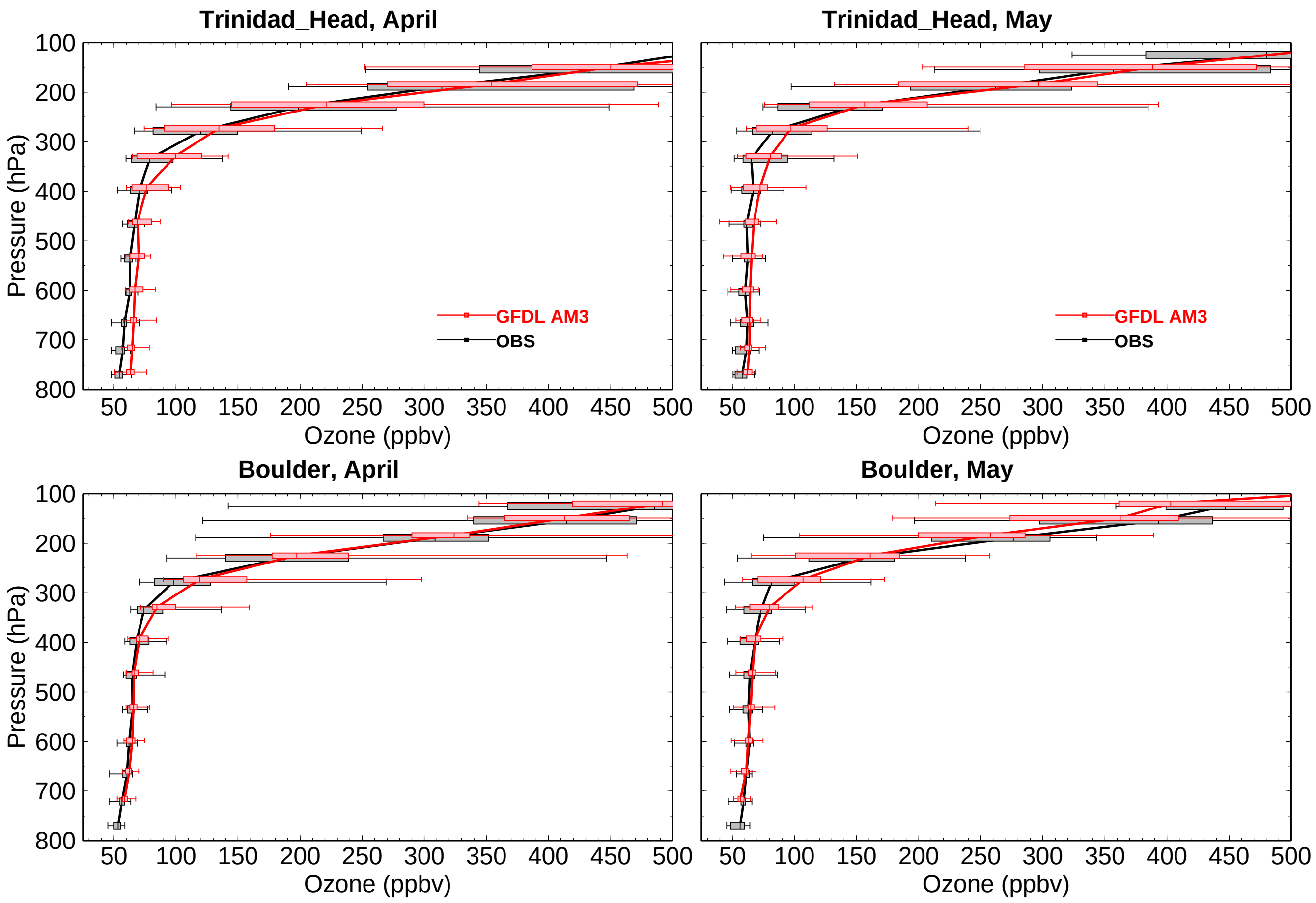

Supplementary Figure 4. **Mean ozone vertical profiles at Trinidad Head and Boulder in April and May**. Shown are observed (black) and simulated (red) results from the GFDL AM3 model. The box-and-whisker plots represent the min, 25th, 50th, 75th, and max of monthly mean values during 1995-2012.

a

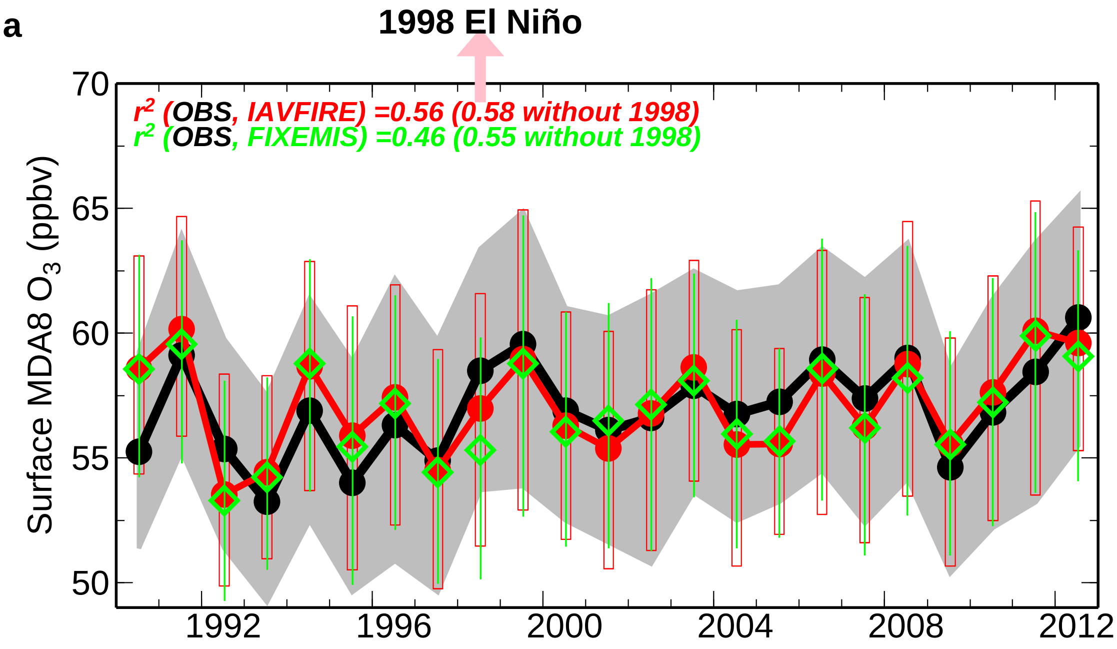

b

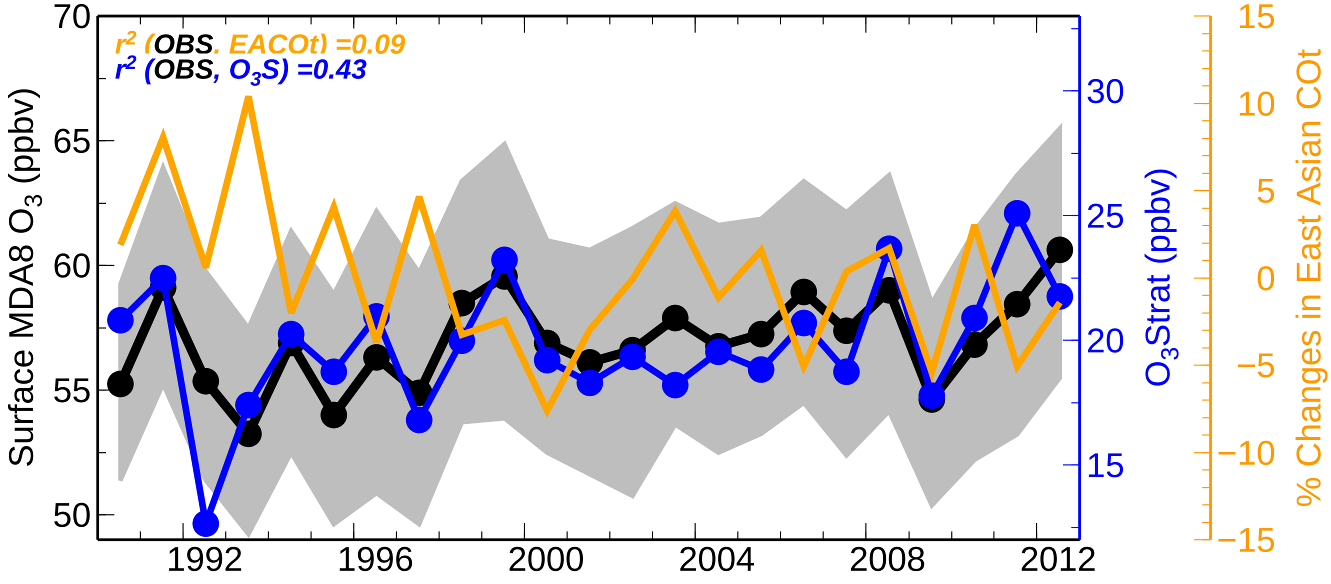

Supplementary Figure 5. **Minor influences from wildfire emissions and Asian pollution on inter-annual variability of western U.S. surface ozone in spring. a**, Surface MDA8 ozone at 22 high-elevation sites in April-May from 1990 through 2012 as observed (black) and simulated by the GFDL AM3 model with inter-annual varying (red, IAVFIRE) versus climatological mean (green, FIXEMIS) emissions from wildfires. The pink arrow denotes the 1998 El Niño event when the largest influence from wildfire emissions occurred. The correlations excluding 1998 are reported in parentheses. **b**, Observed surface MDA8 ozone (black), model tracer of stratospheric ozone (O_3Strat; blue), and a carbon-monoxide-like tracer of East Asian pollution (EACOt; orange) with emissions held constant in time[29]. EACOt is shown as the percentage change from the climatology averaged over the WUS (20-45N; 130-105W).

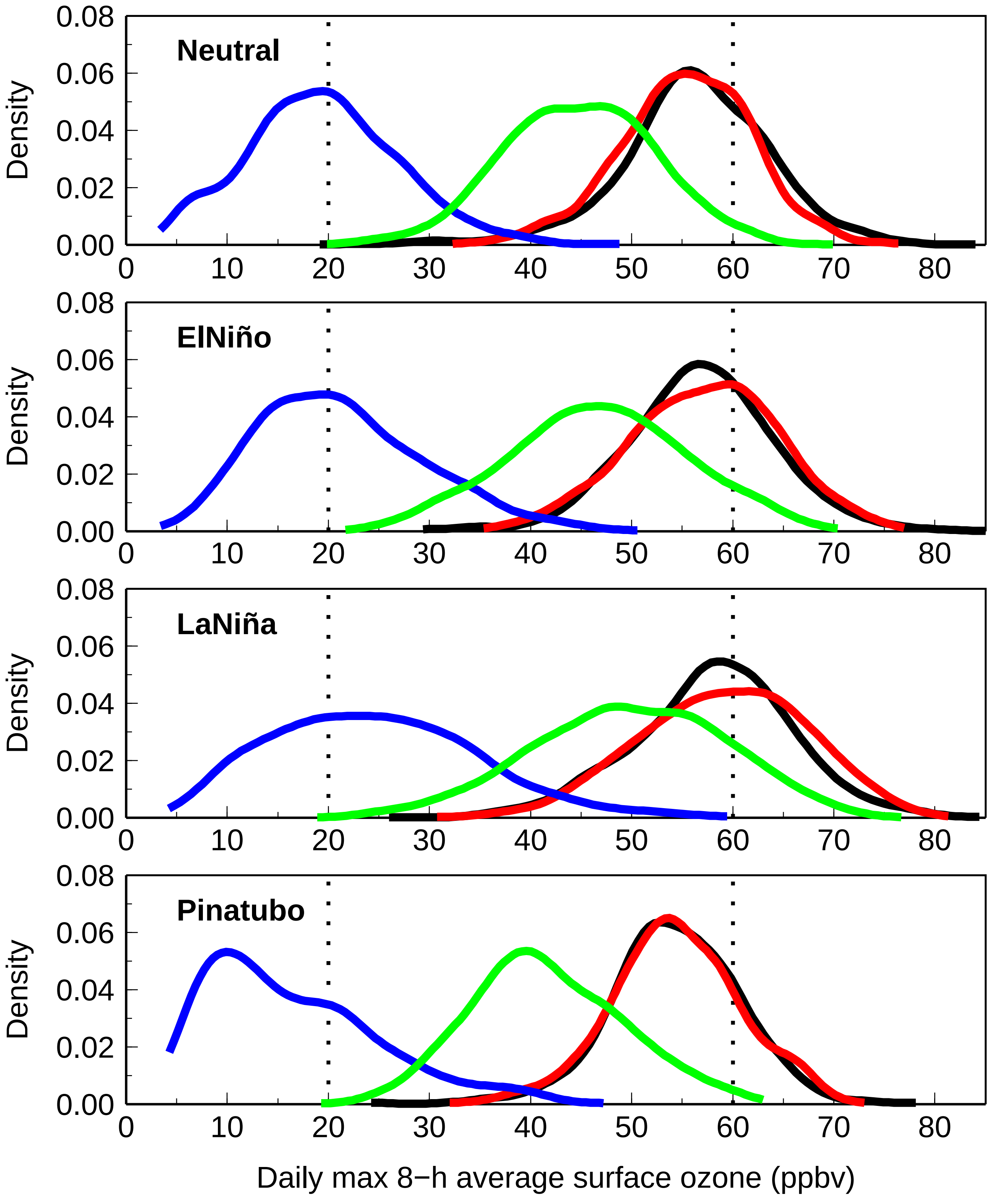

Supplementary Figure 6. Changes in WUS surface ozone distributions related to the different climate states. Probability distribution functions of observed (black) and simulated (red) MDA8 ozone at 22 sites sampled in the model surface level for composites of April and May during Neutral (2004 and 2005), El Niño (1998 and 2010) and La Niña (1999, 2008, 2011) conditions and following the Pinatubo volcanic eruption (1992 and 1993). The stratospheric (blue) and background (green) contributions are shown.

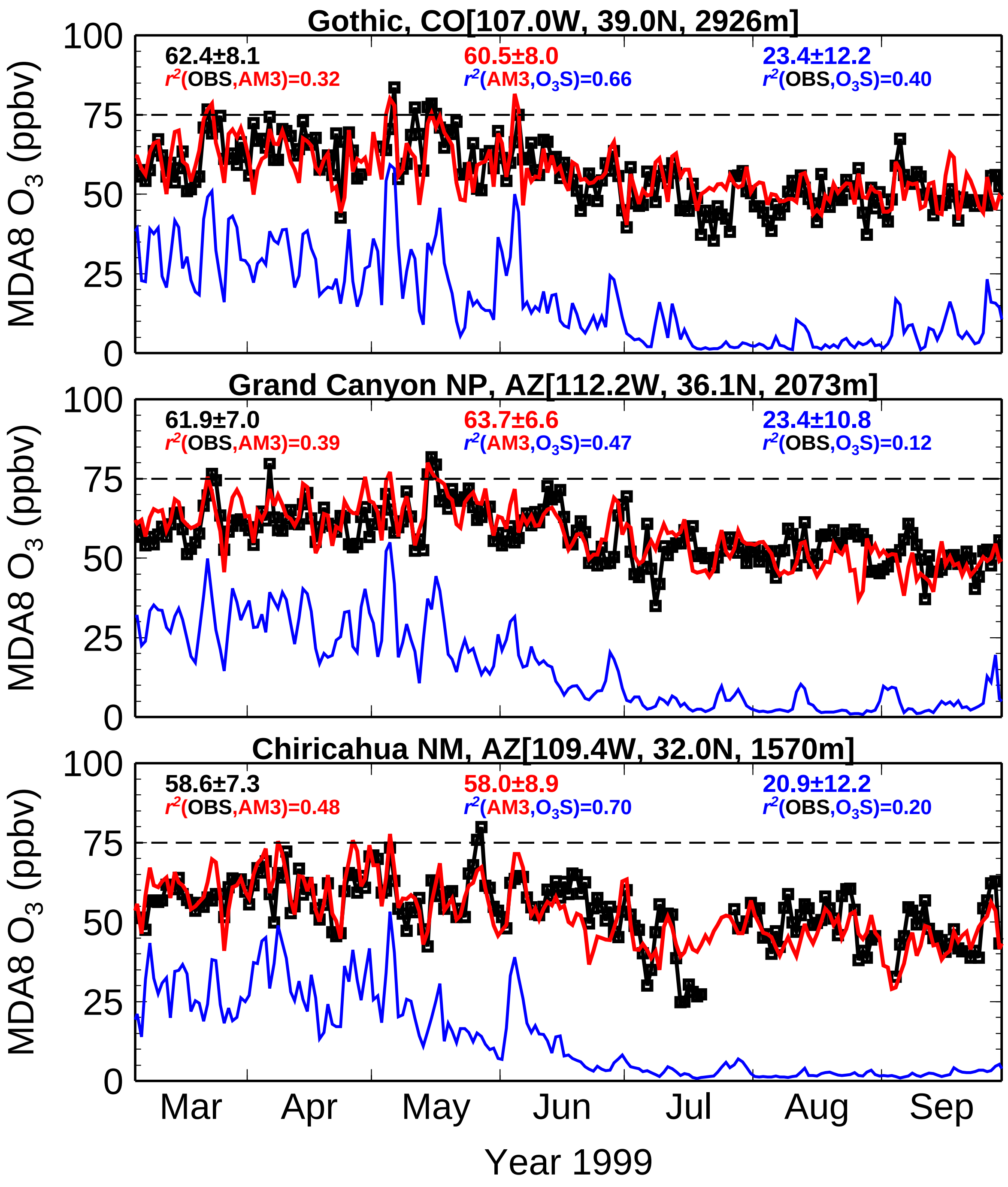

Supplementary Figure 7. More frequent stratospheric intrusions following the 1998-1999 extreme La Niña event. Shown are observed (black) and simulated (red) surface MDA8 ozone in the model surface level from March through September at Gothic in the Colorado Rocky Mountain, Grand Canyon National Park and Chiricahua National Monument in Arizona. Statistics are reported for the April-June period. Blue lines denote the stratospheric contribution. Frequent stratospheric intrusions reaching WUS surface air during spring 1999 are consistent with simulated strong variance in daily O_3Strat at 500 hPa (right column in Supplementary Fig.15).

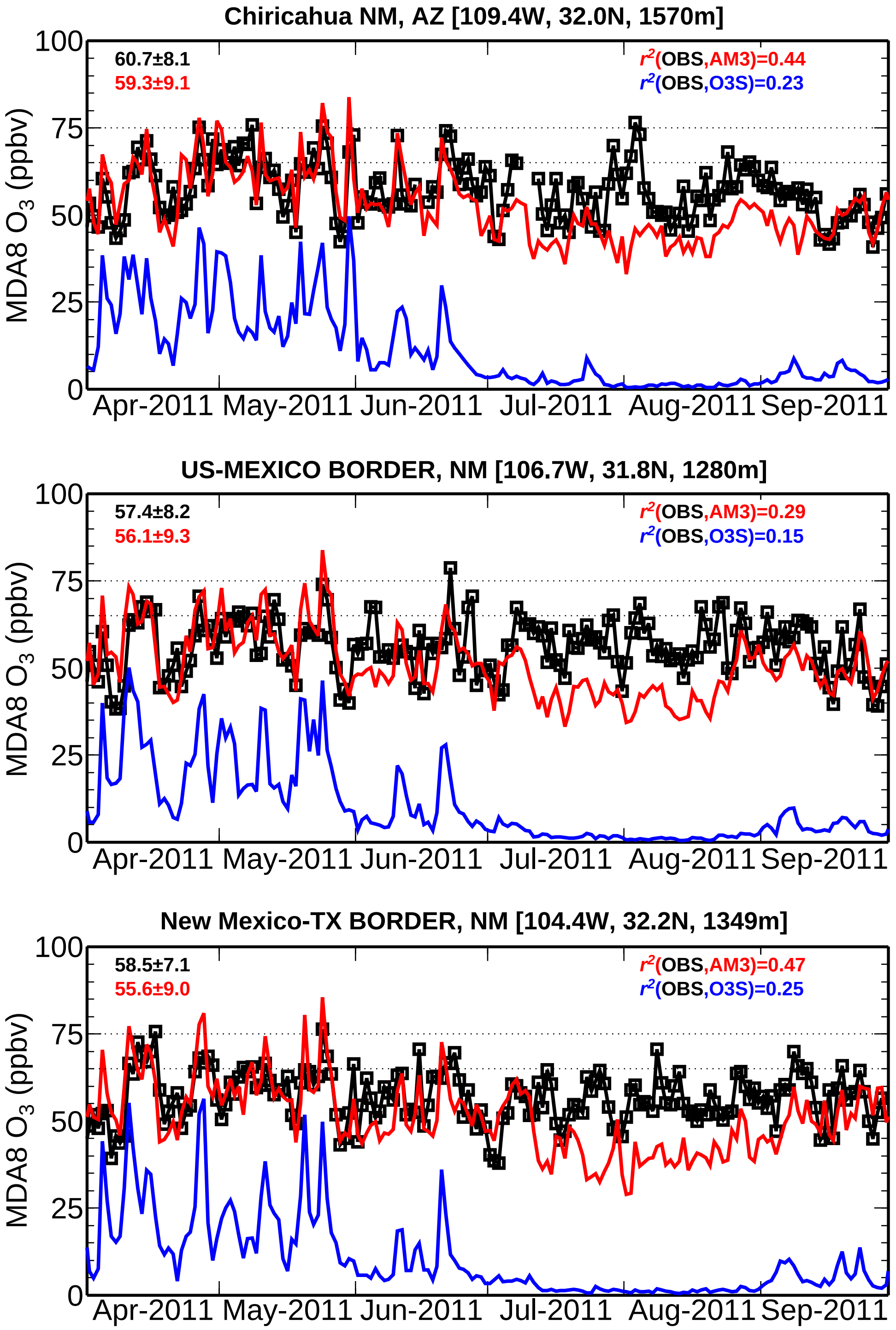

Supplementary Figure 8. Frequent late spring high-ozone events in southwestern U.S. surface air following the strong La Niña winter of 2010-2011. Shown are observed (black) and simulated (red) surface MDA8 ozone from April through June at Chiricahua National Monument in Arizona and two sites near the U.S./Mexico border. The strong stratospheric influence (blue) in southwestern U.S. surface air during spring 2011 is consistent with the southward shift, relative to other La Niña years, in the regions with the greatest variance in daily O_3Strat at 500 hPa (right column in Supplementary Fig.15).

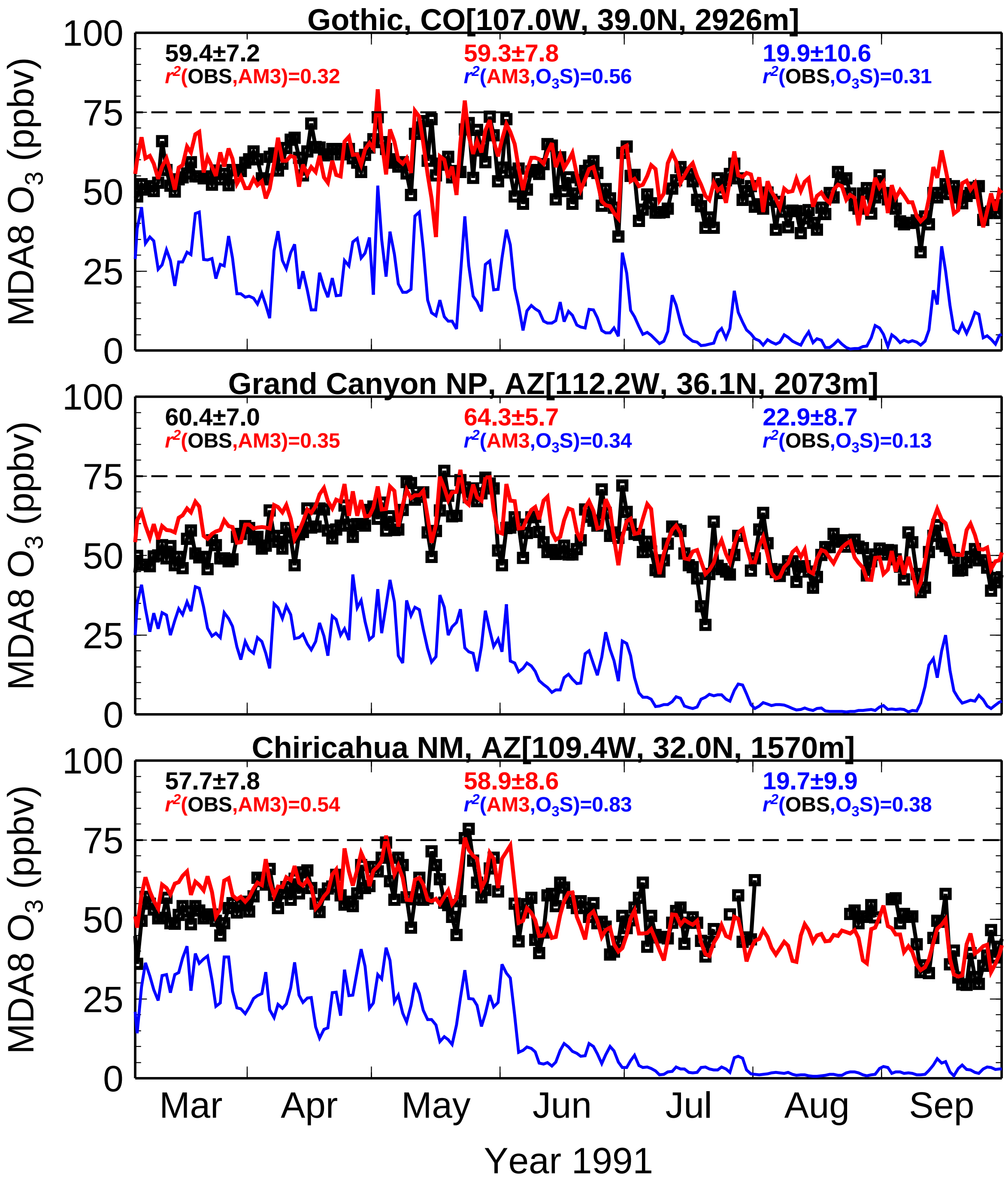

Supplementary Figure 9. **Frequent occurrences of high surface ozone events in April-May 1991**. Shown are observed (black) and simulated (red) surface MDA8 ozone from March through September at Gothic, Colorado, Grand Canyon National Park, and Chiricahua National Monument, Arizona. The stratospheric contribution is shown in blue. Statistics are reported for the April-June period.

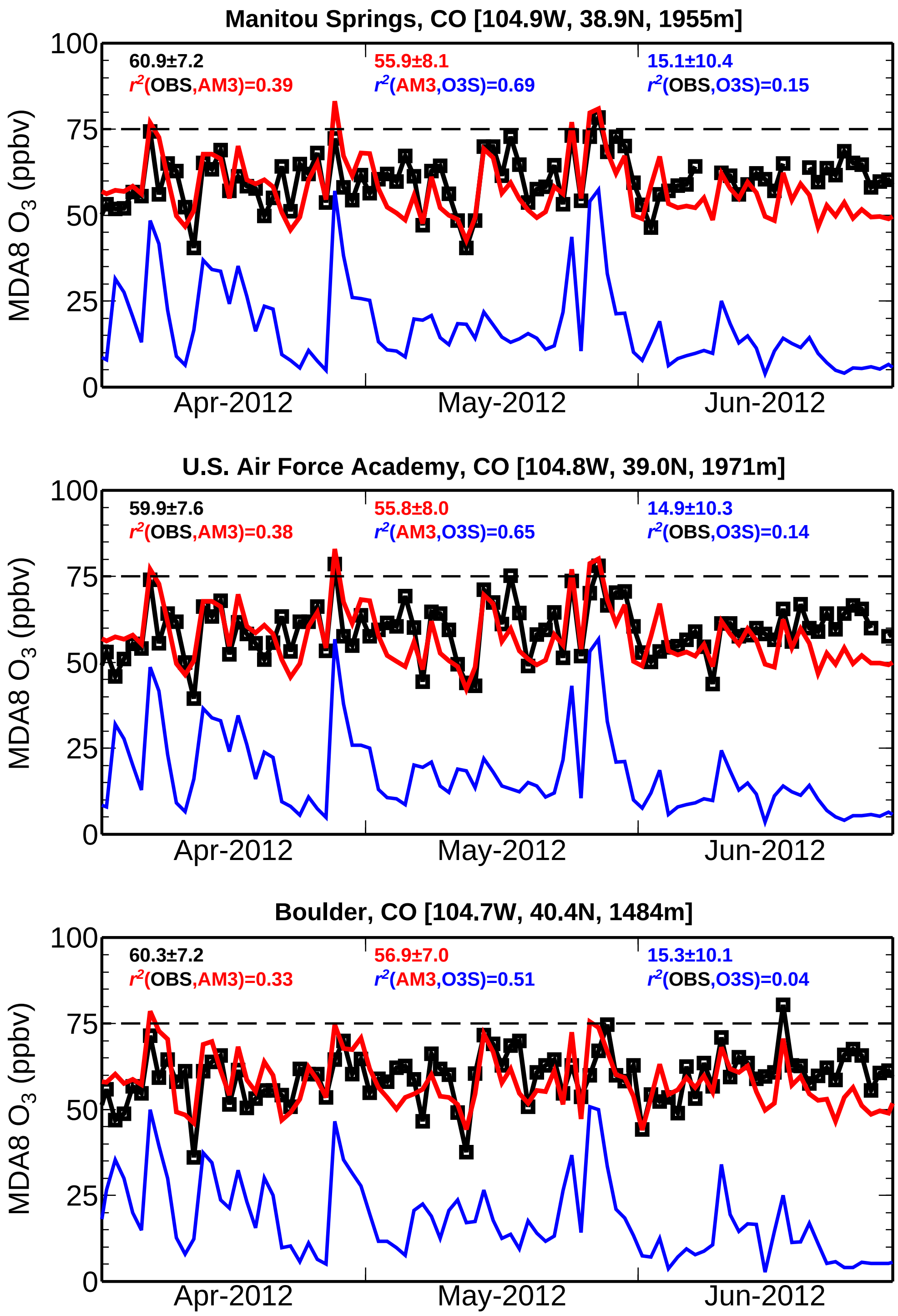

Supplementary Figure 10. **Frequent occurrences of stratospheric intrusions over Colorado during April-June 2012**. Shown are observed (black) and simulated (red) surface MDA8 ozone at Manitou Springs, U.S. Air Force Academy, and Boulder, Colorado. The strong stratospheric influence in surface air (blue) is consistent with the southward dip in the polar jet stream (Supplementary Fig.16c).

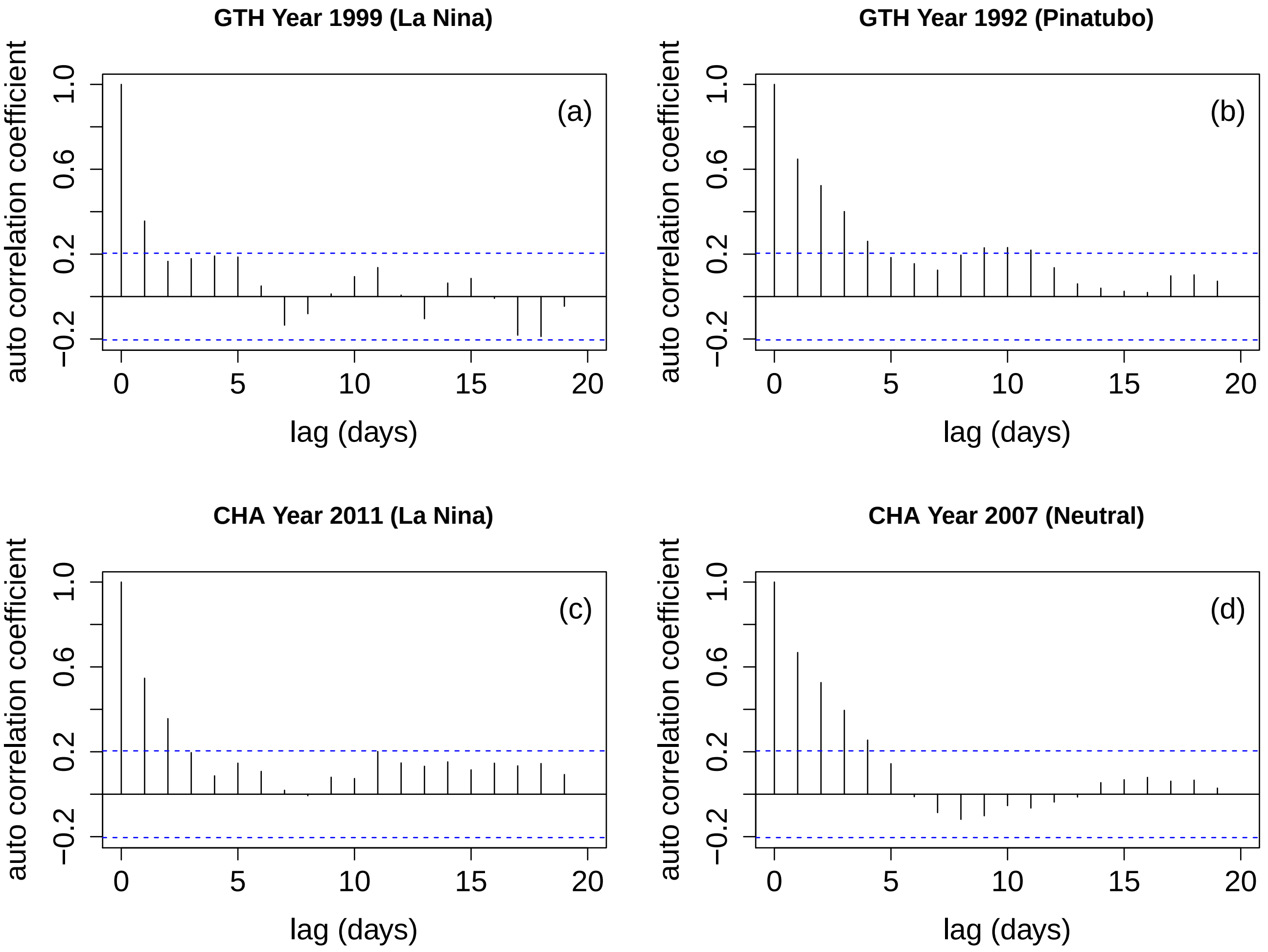

Supplementary Figure 11. **Shorter autocorrelation time scales for daily surface ozone during La Niña springs**. (a-b) The autocorrelation time scales for daily MDA8 ozone at Gothic, Colorado during March through May in 1999 following La Niña versus 1992 following Pinatubo. The blue dashed lines indicate the 95% confidence interval (ci = 1.96/sqrt(sample size)). (c-d) Same as a-b, but for Chiricahua, Arizona in March-May 2011 following La Niña versus 2007 following Neutral conditions.

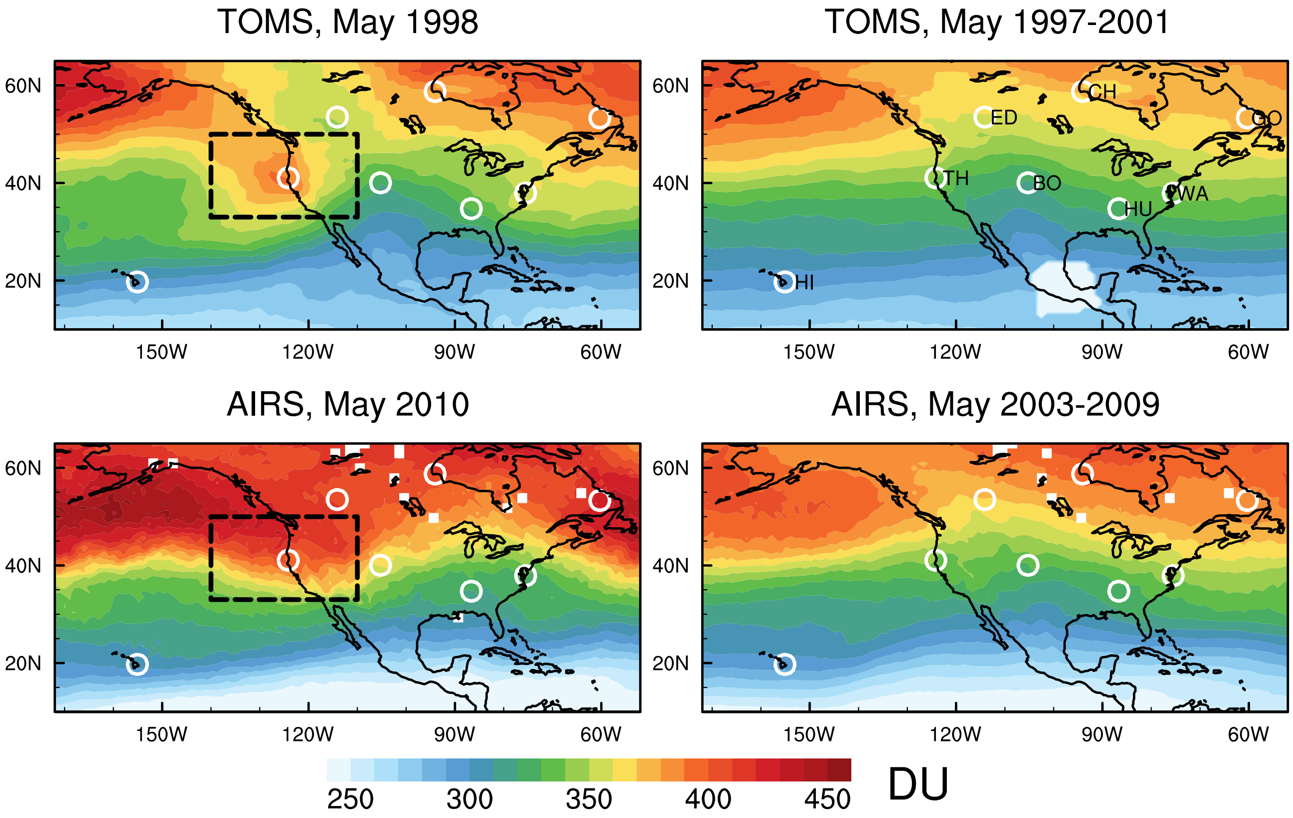

Supplementary Figure 12. Satellite observed enhancements of total ozone columns over Northwest U.S. in May following the strong El Niño winters of 1997-1998 and 2009-2010. (Top) From the NASA Total Ozone Mapping Spectrometer (TOMS) in May 1998 relative to May 1997-2001. (Bottom) From the NASA Atmospheric Infrared Sounder (AIRS) in May 2010 relative to 2003-2009 (Supplementary Note 4). The dashed boxes in the left panels denote where late spring total ozone columns (in Dobson Unit; DU) are enhanced following an El Niño. White circles denote available ozonesonde sites.

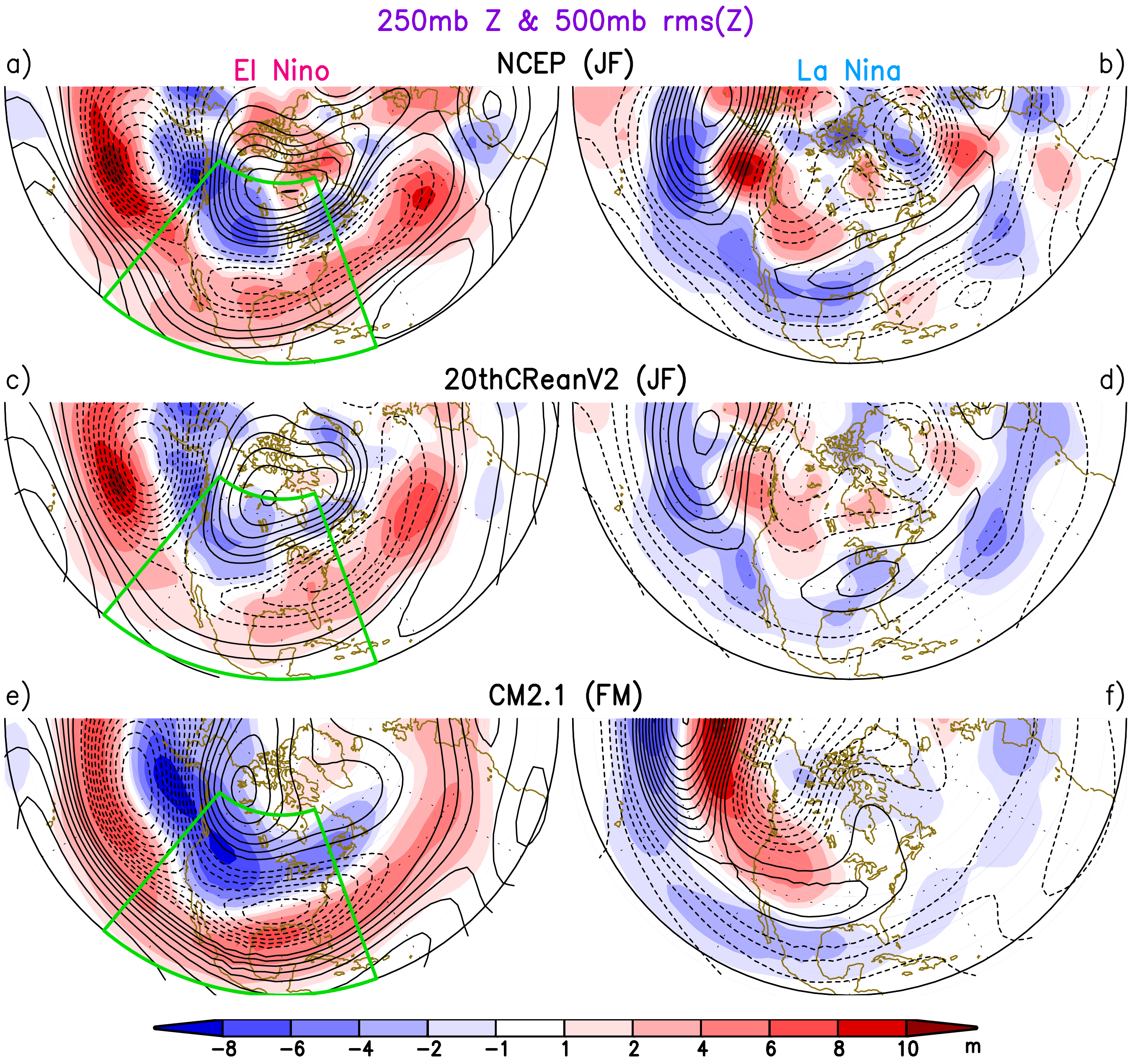

Supplementary Figure 13. **Meridional shifts in late winter mid-latitude storm tracks between El Niño vs La Niña.** Anomalies relative to neutral in the (left) El Niño and (right) La Niña composites of the 250-mb geopotential height (contours: 10m intervals, negative values showned as dashed) and root mean square of the 2-10-day bandpass-filtered 500-mb geopotential height (shading) from: **a-b**, the NCEP-NCAR reanalysis (1948-2008); **c-d**, the twentieth-century reanalysis (1891-2008); and **e-f**, a 2000-year integration of a coupled general circulation model (GFDL CM2.1; without interactive chemistry). Red shading indicates stronger than neutral storm-track intensity (Supplementary Note 3). Reproduced with permission from Supplmentary ref.59.

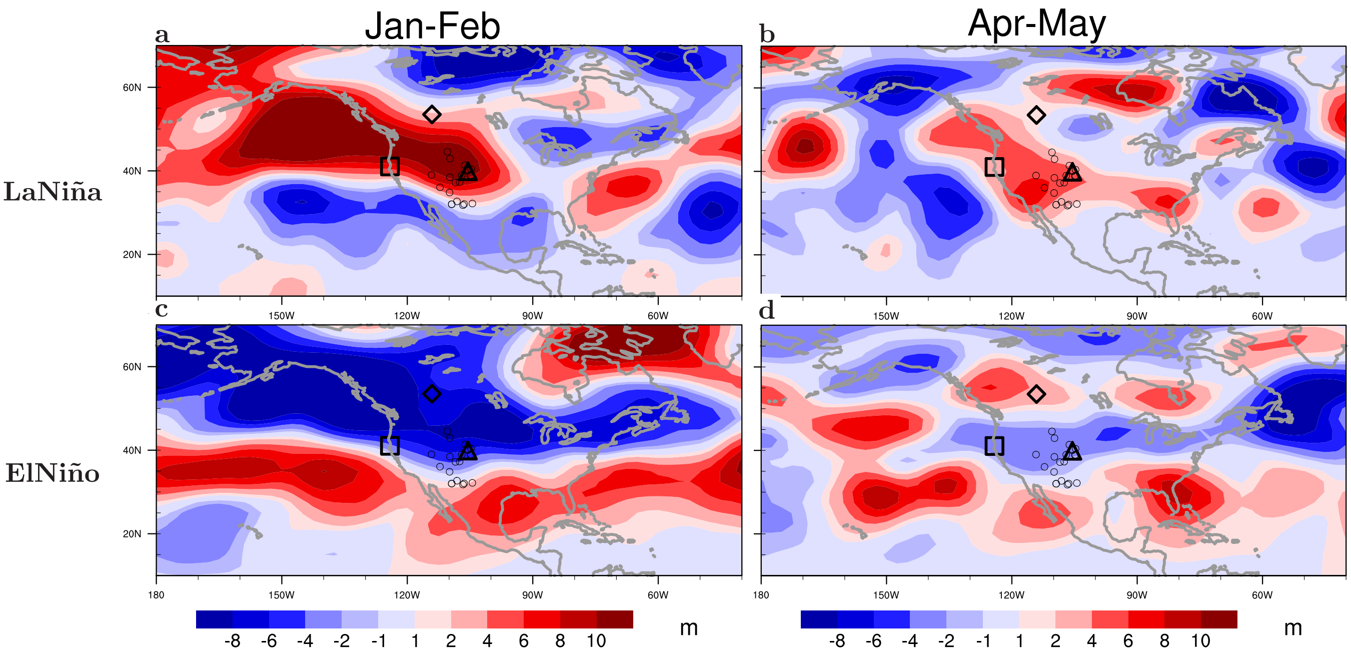

Supplementary Figure 14. **The enhanced storm-track activity over the WUS persists from winter to late spring during strong La Niña events**. Anomalies relative to neutral in the root-mean-square of the 2-10-day bandpass-filtered 500-mb geopotential height in January-February (left) and April-May (right) averaged over all strong La Niña (a-b) and El Niño (c-d) events within the NCEP reanalysis for 1948-2013. Strong ENSO events are defined as the Niño-3.4 index at or above 1.0 for the overlapping 3-month periods of December through April (Methods). Red shading indicates stronger than neutral storm-track intensity (Supplementary Note 3). Symbols denote ozone measurement sites over western North America.

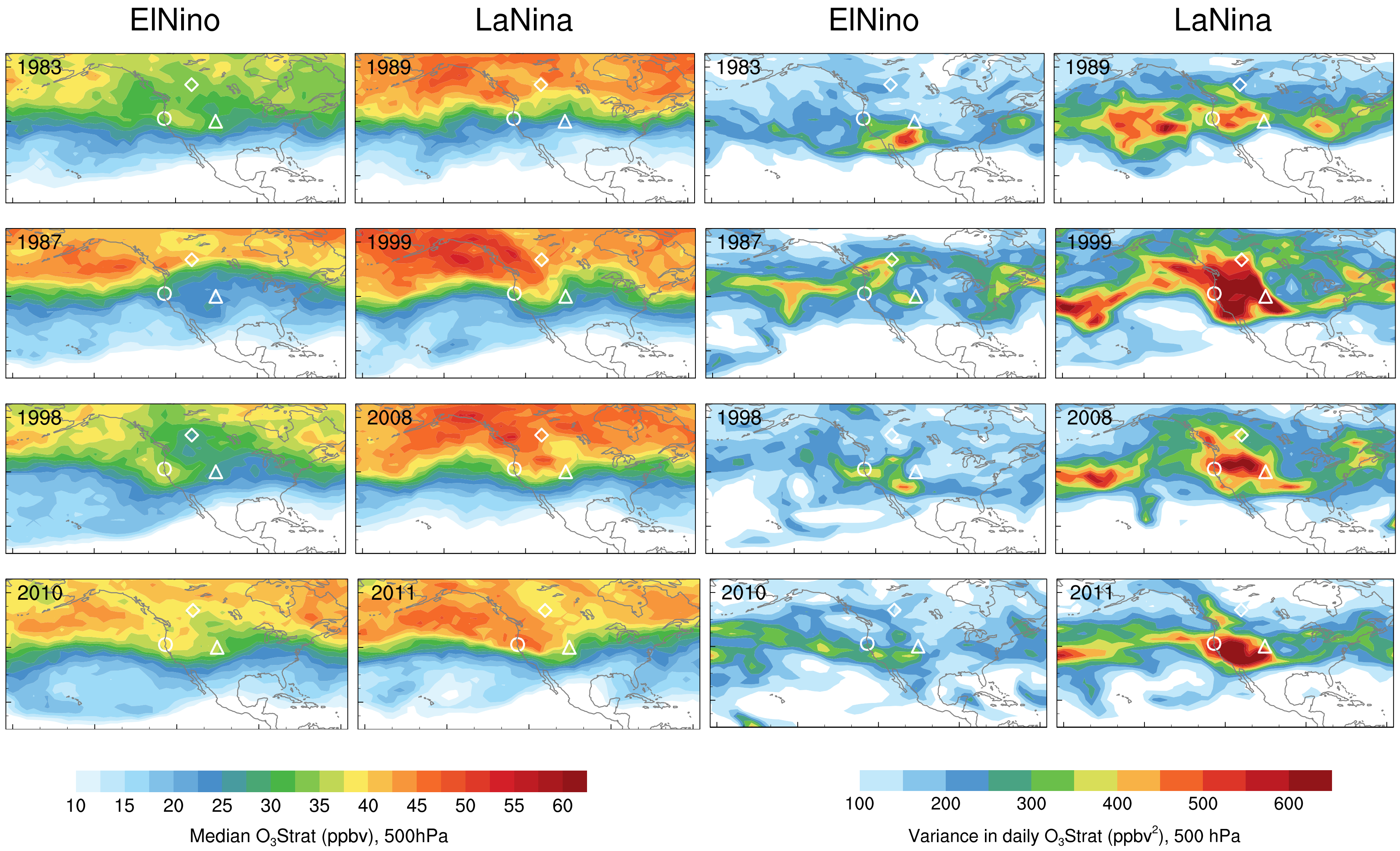

Supplementary Figure 15. **Greater variances of stratospheric influence on WUS mid-tropospheric ozone in each La Niña vs El Niño springs during 1979-2012.** Simulated median and variance of daily stratospheric contribution (O_3Strat) to 500-hPa ozone for each Apr-May following major El Niño (1st and 3rd columns) and La Niña (2nd and 4th columns) events during 1979-2012. The 1991-1992 El Niño is excluded because the El Niño-related ozone change over the WUS is masked by the influence of the Pinabuto volcanic eruption.

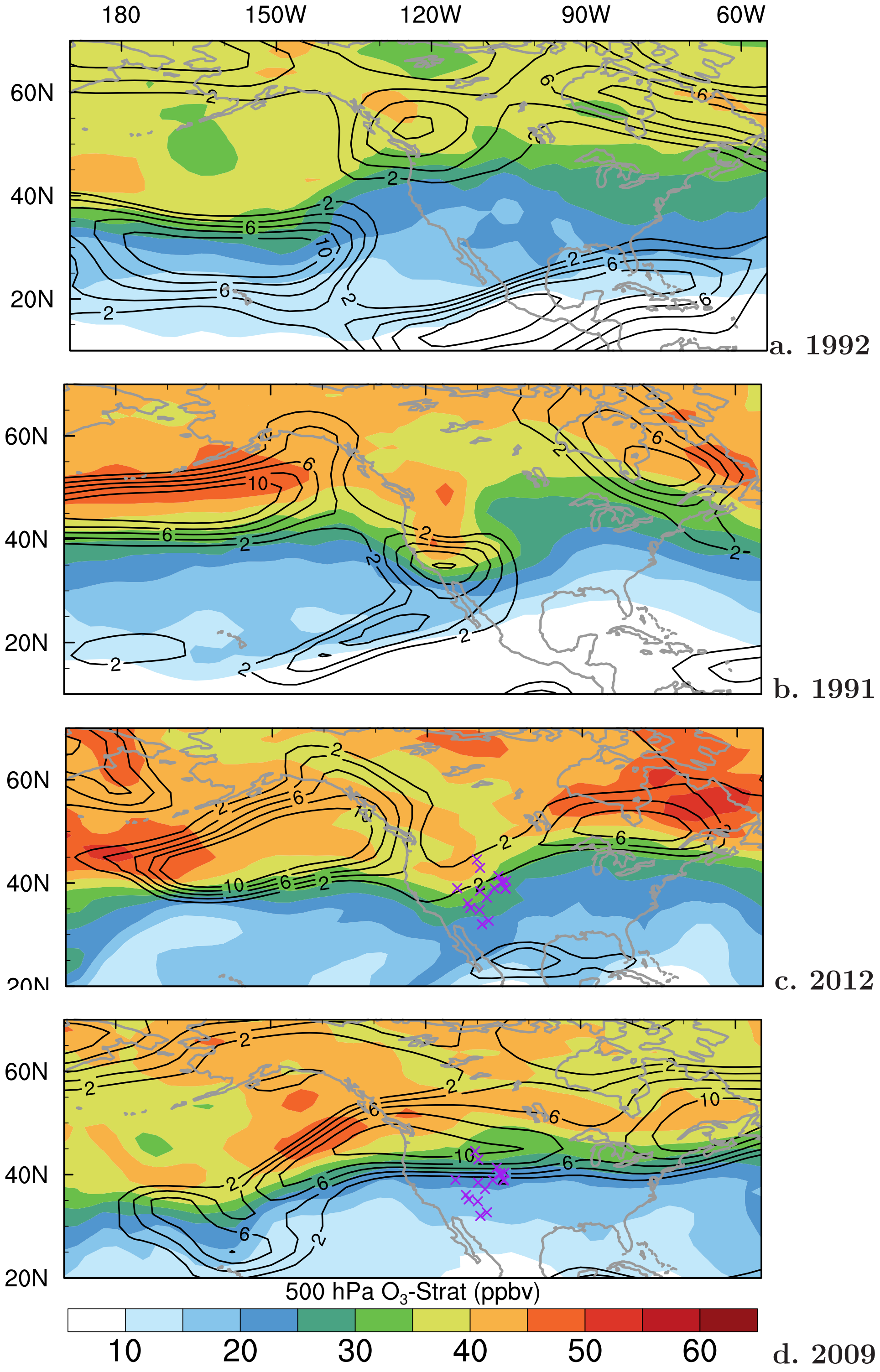

Supplementary Figure 16. **Southward dip in the polar jet stream enables stratospheric ozone transport into the WUS lower troposphere. a-d**, Simulated spatial patterns of stratospheric contribution to 500-hPa ozone (shading) related to jet characteristics (contours) for April-May 1992, April-May 1991, May 2012, and May 2009. Contours indicate 250 hPa wind speed anomalies (from 2 to 10 by every 2 m s^{-1}) relative to climatology, based on the NCEP-NCAR reanalysis. The x symbols indicate the locations of surface monitoring sites.

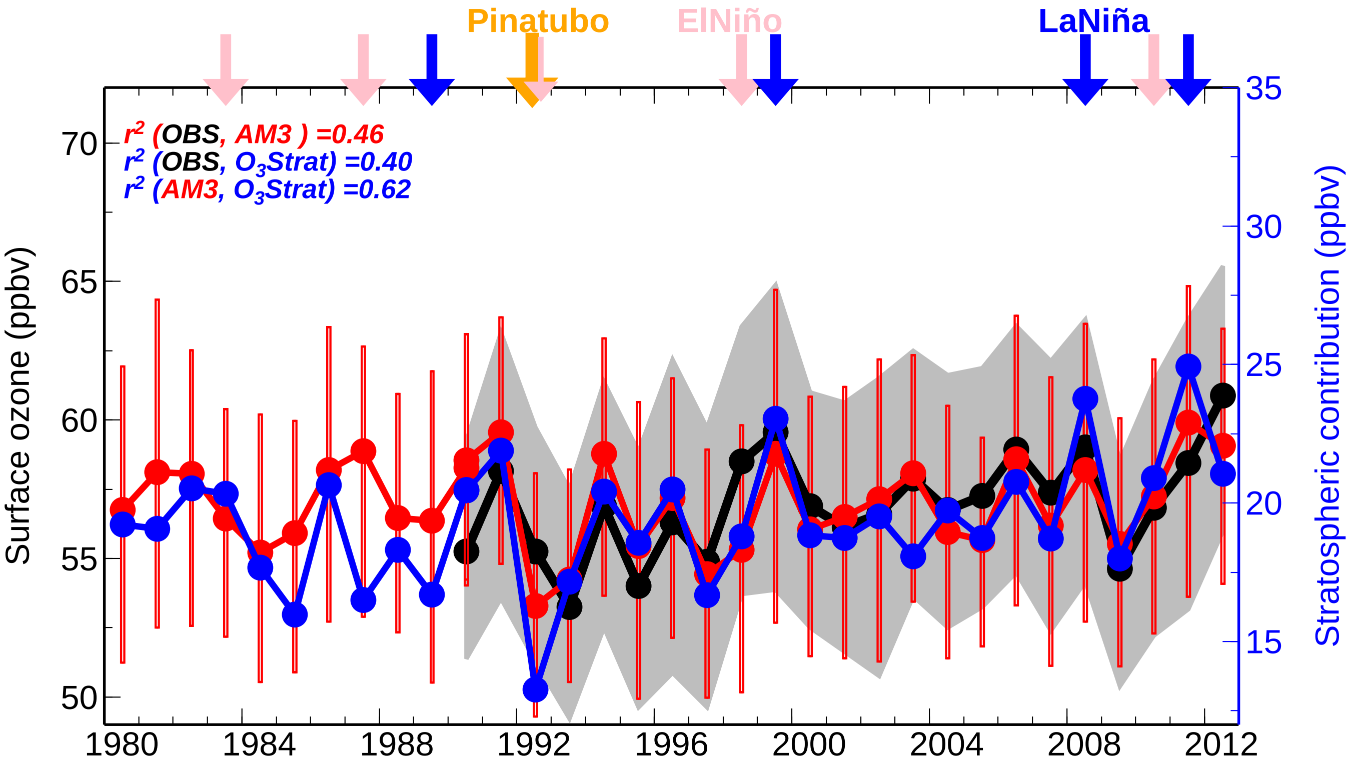

Supplementary Figure 17. **Inter-annual variability of springtime WUS surface ozone tied to stratospheric influence.** Median of maximum daily 8-hour average (MDA8) ozone at 22 high-elevation sites during April-May from 1980 to 2012 as observed (black) and simulated (red) by the GFDL AM3 model with fixed anthropogenic and wildfire emissions; grey shading and red bars, respectively, represent the 25th-75th percentiles. The median stratospheric influence for each year (O_3Strat,blue) is shown using right axis. Arrows at the top of the graph indicate the springs following the Mt Pinatubo volcanic eruption (orange), strong El Niño (pink) and La Niña (blue) winters. The correlation between simulated total ozone and stratospheric contribution is for the entire 1980-2012 period. The lack of covariance between simulated total ozone and stratospheric contribution during April-May of 1987 and 2003 implies that ozone produced from U.S. anthropogenic emissions dominates pollution events in those particular springs.

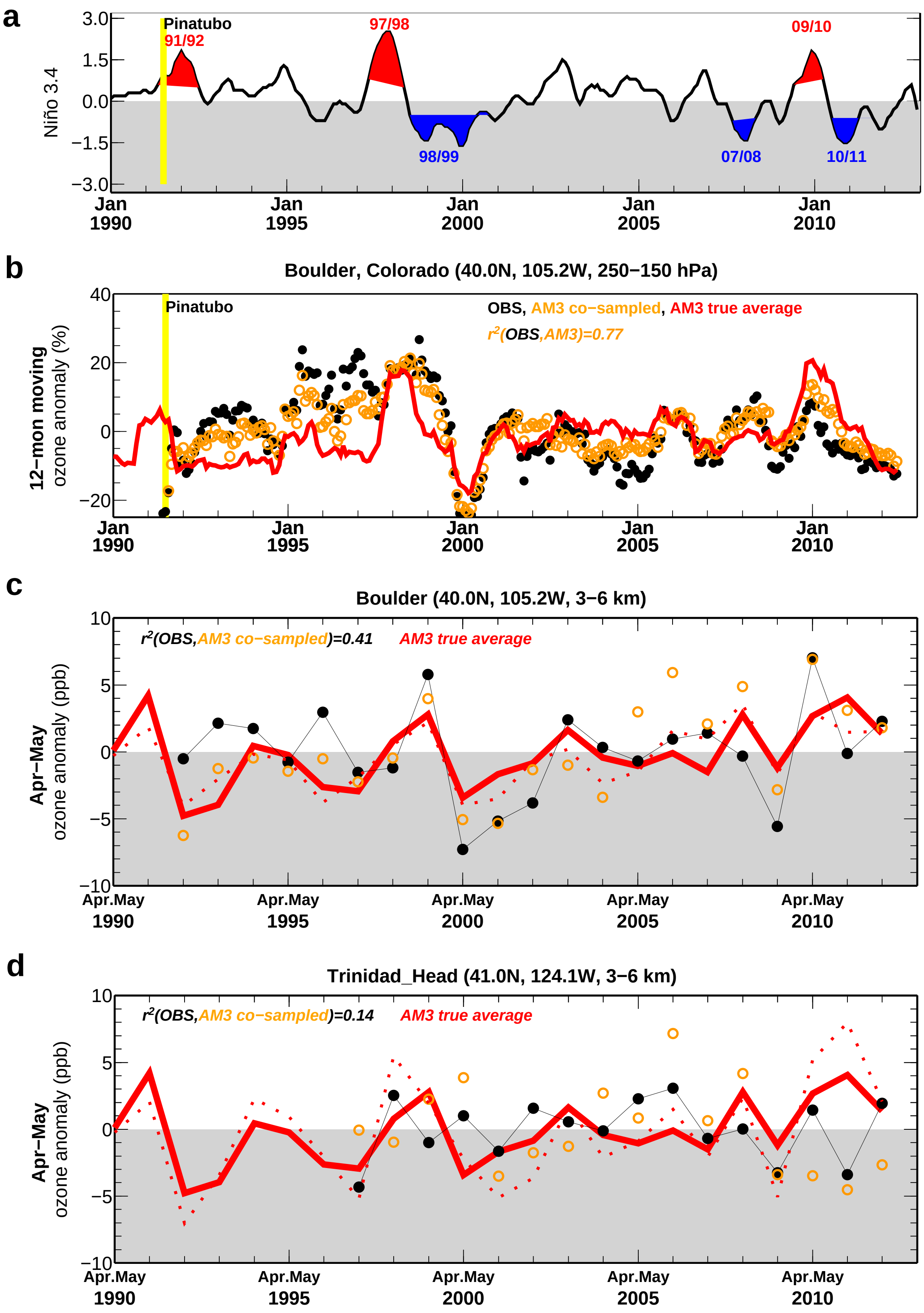

Supplementary Figure 18. **Representativeness of weekly ozonesonde records. a**, The Niño 3.4 index from 1990-2012, highlighting strong El Niño (red) and La Niña (blue) events. **b**, 12-month running mean ozone anomalies (relative to the record mean) over 250-150 hPa at Boulder, Colorado from observations (black), model co-sampled on sonde launch days (orange), model averaged (i.e. unbiased sampling) over all continuous daily ozone fields (red lines). **c-d**, Mean ozone anomalies averaged over 3-6 km altitudes for April-May at Boulder and Trinidad Head, respectively, from observations (black), model co-sampled at sonde launch locations and days (orange), the "true" model average of all days in April-May sampled at the sonde sites (dotted lines) and averaged over the entire WUS domain (box in Fig.7a; thick solid lines).

Supplementary Tables

Supplementary Table 1: Summary of stratospheric impacts on inter-annual variability of tropospheric ozone over northern extratropical regions from previous studies

Reference	Time period	Method	Model treatments of stratospheric O_3 and STT	Summary
**Historical analysis**				
Langford et al. (1998)[1]	1993-1998	Lidar observations / Analysis	N/A	Increased mean UTLS O_3 above Colorado 2-3 months after the 1994-95 (weak) and 1997-98 (strong) El Niños
Langford et al. (1999)[2]	1993-1998	Lidar observations / Analysis	N/A	Correlated variability between mean UTLS O_3 above Colorado and position of the subtropical jet s related to El Niño
Zeng and Pyle (2005)[3]	1990-2001	Tropospheric CCM (3.75ºx2.5º, 19 levels up to 4.6 hPa)	Prescribed stratospheric O_3 above 30 hPa from a 2D model;	Increased global mean STT flux ~6 months after the peak of an El Niño (most evident for 1997-98)
Tarasick et al (2005)[4]	1980-2001	Ozonesonde observation / Analysis	N/A	Correlated mean O_3 variability in the UTLS and in the troposphere at Canadian ozonesonde sites
Thouret et al (2006)[5]	1994-2003	Aircraft observations / Analysis	N/A	Pronounced anomalies in mean UTLS O_3 observed over Atlantic in 1998 and 1999
Hess & Lamarque (2007) [8]	1980-2001	Tropospheric CTM (2.8ºx2.8º, 28 levels up to 2 hPa)	No explicit representation of stratospheric O_3 chemistry; Ozone STT relaxed to a climatological cross-tropopause flux (SYNOZ)	Decreased zonal averaged O_3 north of 50N in spring attributed to a decrease in stratospheric influence associated with a positive AO
Ordonez et al (2007) [6]	1992-2004	Stratospheric CTM (5.6ºx5.6º, 17 levels, 11-55 km altitude);	Lagrangian-based estimates of STT flux	Correlated annual mean O_3 variability in the UTLS and in surface air observed at European alpine sites
Koumoutsaris et al (2008)[7]	1987-2005	Observations / Analysis with Tropospheric CTM (4ºx5º, 30 levels up to 0.01 hPa)	No explicit representation of stratospheric O_3 chemistry; Ozone STT relaxed to a climatological cross-tropopause flux (SYNOZ)	Increased STT influence on mean tropospheric column O_3 over Europe in spring 1998 following the 1997-98 El Niño winter
Terao et al. (2008)[8]	1985-2000	Observations / Analysis with a CTM (2ºx2.5º, 28 levels up to 0.656 hPa)	Explicit stratospheric chemistry; Monthly loss rate in the troposphere	Correlated mean O_3 variability in the UTLS and in the mid-troposphere over northern mid-latitudes
Hsu & Prather (2009)[9]	2001-2005	Tropospheric CTM (T42 ~2.8 degree resolution; 40 levels	LINOZ	Negative anomalies of mid-latitude mean O_3 STT flux during the easterly phases of the QBO
Voulgarakis et al (2011)[10]	1996-2000	Tropospheric CTM (2.8ºx2.8º, 31 levels up to 10 hPa)	Ozone above 100 hPa relaxed to a stratospheric CTM	Increased mean tropospheric O_3 column ~6 months after the peak of 1997-98 El Niño, attributed to changes in dynamics
Cuevas et al. (2012)[11]	1988-2009	Observations / Trajectory analysis	N/A	Increased influences of mid-latitude pollution and stratospheric O_3 at Izana Observatory (28.3N, 16.5W, 2.4 km altitude) during a negative NAO
Pausata et al. (2012)[12]	1980-2005	Tropospheric CTM (2.8ºx2.8º, 31 levels up to 10 hPa)	Stratospheric O_3 relaxed to a monthly mean zonal climatology	Increased mean O_3 over western Europe in winter during a positive NAO/AO
Hess & Zbinden (2013) [12]	1990-2009	Chemistry-climate model driven with re-analysis	No explicit representation of stratospheric chemistry; Ozone STT relaxed to a climatological flux (SYNOZ)	Correlated annual mean O_3 variability at 150 hPa and at 500 hPa averaged over 30-90N latitudes
Tang Q. et al (2013)[13]	1991-1995	Chemistry-climate model	Full stratospheric chemistry	Reduced global mean STT O_3 flux following the Pinatubo eruption
Neu et al (2014)[14]	2005-2010	Satellite measurements of zonal mean ozone above 500 hPa	N.A.	A 2% increase in zonal mean mid-tropospheric O_3 at northern mid-latitudes attributed to variations in STT O_3 flux during the 2009-2010 El Niño vs. the 2007-2008 La Niña.
**Future projections**				
Collins et al (2003)[15]	1990-1994 vs. 2090-2094	Tropospheric CTM (3.75ºx2.5º, 38 levels up to 4.6 hPa)	No explicit representation of stratospheric ozone chemistry in most models	+37% increase in STT O_3 flux due to climate change
Stevenson et al (2006)[16]	2000 vs. 2030	Multi-model CTM/CCM	No explicit representation of stratospheric ozone chemistry in most models, Ozone STT relaxed to a climatology or parameterized (SYNOZ or LINOZ)	+7% increase in global mean STT O_3 flux due to climate change
Hegglin et al. (2009)[17]	1960s vs. 2090s	Stratospheric CCM (6ºx6º, 71 levels up to 100 km)	Chemistry down to 400 hPa; inert below 400 hPa	+23% increase in global mean STT O_3 flux due to climate change
Zeng et al (2010)[18]	2000 vs. 2100	Tropospheric CCM (3.75ºx2.5º, 19 levels up to 4.6hPa)	Prescribed O_3 in the lower stratosphere	+43% increase in global mean STT O_3 flux due to climate change and stratospheric O_3 recovery

Supplementary Table 2: List of Western U.S. surface ozone monitoring sites

State	Site Name	Latitude	Longitude	Elevation (m)	Period	Operation Agency	Network Site Abbreviation
Arizona	Chiricahua NM	32.01	-109.39	1570	1992-2012	NPS/CASTNET	CHA467
Arizona	Chiricahua NM Collocated	32.01	-109.39	1570	1990-1992	EPA/CASTNET	CHA267
Arizona	Grand Canyon NP	36.06	-112.18	2073	1990-2012	NPS/CASTNET	GRC474
Arizona	Petrified Forest	34.82	-109.89	1723	2003-2012	NPS/CASTNET	PET427
Colorado	Rocky Mountain NP	40.28	-105.55	2743	1991-2012	EPA/CASTNET	ROM406
Colorado	Gothic	38.96	-106.99	2926	1990-2012	EPA/CASTNET	GTH161
Colorado	Mesa Verde NP	37.2	-108.49	2165	1994-2012	NPS/CASTNET	MEV405
Colorado	Manitou Springs	38.85	-104.9	1955	2004-2012	EPA/AQS	08-041-0016
Colorado	U.S. Air Force Academy	38.96	-104.82	1971	1997-2012	EPA/AQS	08-041-0013
Colorado	Niwot Ridge C1	40.04	-105.54	3022	1991-1998<br>2003-2012	NOAA/GMD	NWR
Colorado	Niwot Ridge Tundra Lab	40.05	-105.59	3538	2003-2012	NOAA/GMD	TUN
Colorado	Boulder	40.38	-104.74	1484	2003-2012	EPA/AQS	08-123-0009
Nevada	Great Basin NP	39.01	-114.22	2060	1994-2012	NPS/CASTNET	GRB411
New Mexico	Silver City	32.69	-108.12	1823	2005-2012	EPA/AQS	35-017-1003
New Mexico	U.S. Mexico Border	32.18	-104.44	1349	1996-2011	EPA/AQS	35-015-3001
New Mexico	U.S. Mexico Border	32.04	-106.41	1250	2001-2011	EPA/AQS	35-013-0020
New Mexico	U.S. Mexico Border	31.79	-106.68	1280	2001-2011	EPA/AQS	35-013-0022
Utah	Canyonlands NP	38.46	-109.82	1809	1993-2012	NPS/CASTNET	CAN407
Wyoming	Pinedale	42.93	-109.79	2388	1990-2012	EPA/CASTNET	PND165
Wyoming	Centennial	41.36	-106.24	3178	1992-2012	EPA/CASTNET	CNT169
Wyoming	Yellowstone NP	44.56	-110.4	2400	1997-2012	NPS/CASTNET	YEL408
Wyoming	Lake Yellowstone	44.56	-110.39	2361	1991-1996	NPS/CASTNET	YELL-LV

Supplementary Table 3: Statistics of Western U.S. surface ozone measurements in April-May

**Year**	**Total MDA8 samples**	**# samples with MDA8 ≥ 75 ppb**	**% samples with MDA8 ≥ 75 ppb**
1990	349	4	1.1
**1991**	**447**	**7**	**1.6**
**1992**	**462**	**3**	**0.6**
**1993**	**528**	**0**	**0**
1994	649	4	0.6
1995	602	0	0
1996	540	11	2.0
1997	*617*	*0*	*0*
**1998**	**698**	**7**	**1.0**
**1999**	**602**	**20**	**3.3**
2000	635	2	0.3
2001	752	1	0.1
2002	754	7	0.9
2003	841	5	0.6
2004	1082	10	0.9
2005	1166	4	0.3
2006	1172	12	1.0
2007	1183	7	0.6
**2008**	**1142**	**15**	**1.3**
2009	1171	2	0.2
**2010**	**1200**	**18**	**1.5**
**2011**	**1012**	**25**	**2.5**
**2012**	**1030**	**26**	**2.5**

Supplementary Table 4: Fritz Peak ozonelidar and Boulder ozonesonde data

Year	Fritz Peak, Colorado (39.9N, 105.5W)		Boulder ozonesonde, Colorado (40.0N, 105.20W)	
	# of daily profiles	# of samples (6.0±0.8 km altitude)	# of daily profiles	# of samples (4-6 km altitude)
Apr-May 1994	18	90	8	160
Apr-May 1995	13	65	5	100
Apr-May 1996	17	85	6	120
Apr-May 1997	12	60	9	180
Apr-May 1998	18	90	5	100
Apr-May 1999	12	60	8	160

Supplementary Table 5: Summary of forcings and emissions used in GFDL AM3

Experiment	Period	Radiative forcings	CH_4 (chemistry)	Anthropogenic emissions of aerosol and ozone precursors	Fire Emissions
FIXEMIS	1979-2012	Historical	2000	1970-2010 climatology	1970-2010 climatology
IAVFIRE	1979-2012	Historical	2000	1970-2010 climatology	Historical
BACKGROUND	1979-2012	Historical	Historical	Shut off in North America; Historical elsewhere	Historical

Supplementary Notes

Supplementary Note 1: Review of Previous Studies

The stratospheric influence on inter-annual variability of tropospheric ozone over extratropical regions has been examined through observational and modeling studies focusing on relationships to known modes of climate variability (e.g. El Niño-Southern Oscillation - ENSO[1-3,7,10,14], Arctic Oscillation - AO[11,12,19,20], and Quasi-Biennial Oscillation - QBO[9,14]), correlations between annual mean ozone changes in the troposphere versus in the lower stratosphere[4,6,8,21], and increases under future climate scenarios[15-18]. These studies (summarized in **Supplementary Table 1**) suggest that the large-scale subsidence of stratospheric ozone exerts a considerable influence on the inter-annual variability of the mean tropospheric ozone burden in northern extratropical regions, with the largest impact occurring in the middle to upper troposphere during winter and spring[22]. The influences of ENSO on deep convection over the tropical Pacific and associated tropospheric ozone variability have also been noted[23,24]. Much less attention has been paid to the mechanisms controlling the frequency and intensity of deep stratospheric intrusions reaching the lower troposphere, which have been found to be most active over the high-elevation western U.S. during spring[19,25]. Recent observational and modelling analysis indicates that deep stratospheric intrusions can episodically push observed surface ozone at western U.S. (WUS) high-elevation sites to exceed the 75 ppb level of U.S. air quality standard for ground-level ozone[26-31]. These intrusions can be classified as “exceptional events”, which do not count towards regulatory decisions[32-36].

Ozone changes in the lower stratosphere can be induced by changes in stratospheric circulation, anthropogenic chlorine, and volcanic aerosols. For example, the combined effects of anthropogenic chlorine and volcanic aerosols have resulted in observed record-low levels of ozone in the lower stratosphere following the 1991 eruption of Mt Pinatubo in the Philippines[13,37-39]. Low ozone anomalies were also observed in the troposphere during 1992-1993 at some northern high-latitude (>45ºN) sites[40,41]. Models with a climatological stratosphere are unable to capture the 1992-1993 ozone anomalies[7,21], suggesting that a key source of the observed variability involves coupled stratosphere-troposphere chemical and meteorological processes.

High ozone anomalies have been observed in the upper troposphere and lower stratosphere (UTLS) at some Arctic and mid-latitude sites in late winter and spring 2-4 months after moderate to strong El Niño conditions in the tropical Pacific Ocean[1,14,42]. Despite early failed attempts to identify a significant

ENSO signal in the stratosphere[43], recent climate modeling studies have attributed the increased ozone in the Arctic and mid-latitude lower stratosphere to variability in atmospheric circulation through stratosphere-to-troposphere coupling during El Niño and/or the easterly shear QBO in the tropical lower stratosphere[14,42,44,45]. Namely, more upward propagating planetary wave activity reaches the stratosphere during El Niño[42], which diminishes the strength of the polar vortex[46] and strengthens the meridional circulation in the middle stratosphere[44,47,48].

A few tropospheric chemical transport models (CTM), which have poorly resolved stratospheres and often use prescribed stratospheric ozone, indicate an increase in global mean stratosphere-to-troposphere (STT) ozone flux 4-6 months after the major El Niño event of 1997-98 (refs. [3,7,10]; **Supplementary Table 1**). The extent to which these interannual variations in UTLS ozone burdens, in the global mean STT flux, or in zonal mean mid-tropospheric ozone reach the surface - relevant for regional air quality - is poorly characterized.

The stratospheric intrusions that penetrate deeper into the troposphere have an episodic, transient and localized nature[49]. They typically appear as fine-scale filamentary structures identified in a diverse suite of observations. For example, they have long been detected by aircraft measurements showing layers with enriched ozone with a deficit in carbon monoxide and water vapor[30,50-52]. They are evident in ozone lidar measurements and in meteorological tracers like potential vorticity[28,29,53,54]. They appear as unusually dry layers with high ozone concentrations in soundings[29,55,56].

A recent study shows that deep STT mass flux into the boundary layer during spring is a factor of 4 greater over the WUS than other northern mid-latitude regions[25] (see their Fig.5). Transport of stratospheric ozone to the WUS surface is mainly associated with deep tropopause folds that form in upper-level frontal zones below the polar jet stream[25,27,29,30]. Strong La Niña episodes in the tropical Pacific are known to have distant effects on the frequency and intensity of cold frontal passages over northwestern U.S. regions and position of the polar jet stream over the U.S. and Canada, particularly during winter[57-60]. The extent to which these La Niña-related storm track shifts affect deep tropopause folds and surface ozone variability over the WUS has not been examined.

Advancing knowledge on how climate variability modulates the stratospheric influence on lower tropospheric ozone necessitates a fully coupled chemistry-climate model, which can represent the interplay among stratosphere-troposphere chemistry and dynamics on daily to interannual time scales. Here we use such a model and observations to identify the underlying mechanisms controlling the frequency of springtime high-ozone episodes observed at the WUS surface.

Supplementary Note 2: Additional surface ozone time series analysis

Spring 1999: Following the strong La Niña winter of 1998-1999, frequent stratospheric intrusions led to high-ozone events observed at surface sites across the Western U.S. intermountain regions during spring 1999. **Supplementary Figure 7** shows the examples for Gothic in Colorado, Grand Canyon National Park in Arizona, and Chiricahua National Monument in Arizona. Both observed and simulated day-to-day fluctuations in surface MDA8 ozone decrease substantially from spring to summer months, consistent with known seasonality of stratospheric influence. The strong variance in daily O_3Strat at 500 hPa (right column in **Supplementary Fig.15**) further supports the conclusion that observed high-ozone events in WUS surface air are associated with deep stratospheric intrusions.

Spring 2011: Following the strong La Niña winter of 2010-2011, frequent stratospheric intrusions occurred during the late spring to early summer of 2011. These intrusion events have the greatest impact on the southwestern U.S. surface regions. **Supplementary Figure 8** shows the examples for Chiricahua National Monument in Arizona and two sites near the U.S./Mexico border. The strong stratospheric influence in southwestern U.S. surface air during spring 2011 is consistent with the southward shift, relative to other La Niña springs, in the regions with the greatest variance in daily O_3Strat at 500 hPa (right column in **Supplementary Fig. 15**).

Spring 1991: Frequent stratospheric intrusions occurred during the late spring of 1991 when the polar jet meanders towards the southwestern U.S., in sharp contrast to 1992 and 2009 (**Supplementary Fig.16b**). High-ozone events above 65 ppb, coincident with increasing stratospheric influence in the model, were observed at Gothic, Colorado, Grand Canyon National Park, Arizona, and Chiricahua National Monument, Arizona during April-May 1991 (**Supplementary Figure 9**)

Spring 2012: Anomalously frequent high-ozone events were also measured at WUS high-elevation sites during spring 2012 (**Supplementary Table 3**). **Supplementary Figure 10** shows time series analysis of surface ozone at Manitou Springs, U.S. Air Force Academy, and Boulder in Colorado. The AM3 O_3Strat tracer increases by 30-40 ppb (above the baseline level) on days when observed MDA8 ozone exceeds the 75 ppb NAAQS level (e.g. April 6, April 27, and May 27), indicating the influence from deep stratospheric intrusions. The strong stratospheric influence is consistent with the southward dip in the polar jet stream (**Supplementary Fig.16c**), which facilitates deep tropopause folds.

Supplementary Note 3: ENSO and mid-latitude storm track characteristics

Supplementary Figures 13 and 14 illustrate mid-latitude storm track characteristics during the warm El Niño vs. cold La Niña phases of ENSO using eddy variance and covariance statistics. A 2-10-day bandpass Lanczos filter with 41 weights is applied to the daily 500-hPa geopotential height field as described in Li and Lau (2012)[59], and then anomalies (relative to neutral) in the root-mean-square (rms) of the filtered height for January-February and April- May is constructed by averaging over all strong El Niño and La Niña events. The rms field is a good indicator of the intensity and location of the storm tracks[57].

The comparison of winter and spring flow patterns **(Supplementary Fig.14)** focuses on the strong ENSO events defined as the Niño 3.4 index at or above +/- 1.0ºC anomaly for the overlapping 3-month periods of Dec-Jan-Feb, Jan-Feb-Mar, and Feb-Mar-Apr. By this definition, the 66 years of the NCEP reanalysis include the strong El Niño events of 1957-1958, 1965-1966, 1968-1969, 1982-1983, 1986-1987, 1991-1992, 1997-1998, and 2009-2010, and the strong La Niña events of 1970-1971, 1973-1974, 1988-1989, 1998-1999, 2007-2008, and 2010-2011.

The wintertime responses of the storm track and regional climate over the North Pacific and North America to ENSO events have been well documented[57-60]. The spatial distributions of storm-track intensity show distinct meridional shifts in the zone of maximum transient eddy activity over southern United States–Gulf of Mexico during El Niño, in contrast to over the U.S. Pacific Northwest region during La Niña[59] (see red shading in **Supplementary Fig.13**). These findings are consistent with the results of Seager et al[60], who showed that transient eddies propagate along a more southern path towards southwestern North America during El Niño, while they take a more northward route towards the U.S. Pacific Northwest during La Niña.

The springtime responses of atmospheric circulation patterns over the North Pacific and North America to ENSO events are less certain**.** We find that the enhanced storm-track activity over the central western U.S. extends from winter into late spring during strong La Niña events (**Supplementary Fig.14**). Despite the weakened storm-track activity in April-May compared to January-February, the greater cross-tropopause ozone flux in late spring[61] amplifies the signals of ENSO in ozone. We show that the variance in daily O_3Strat in the western U.S. free troposphere is a factor of two greater during La Niña than El Niño springs (**Supplementary Fig.15**), reflecting stronger deep tropopause folds at the polar frontal jet than at the subtropical jet. It is known that variations in atmospheric circulation patterns

during each ENSO event are somewhat different from another due to atmospheric noise[62]. During the springs following the strong El Niño events of 1982-83 and 1997-98, the zone of maximum variance in daily O_3Strat occurs over southern United States–Gulf of Mexico (3rd column in **Supplementary Fig.15**), consistent with the zone of maximum storm track intensity associated with El Niño[59]. Overall, both the composition analysis (**Fig. 7**) and the comparison for individual events during the past 34-year period (3rd vs. 4th column in **Supplementary Fig.15**) indicate that the springtime variance in daily O_3Strat at 500 hPa over the WUS is greater following a La Niña winter than El Niño.

Supplementary Note 4: Additional Information for Ozone Measurement Data

Surface Ozone Measurements: **Supplementary Table 2** gives names and locations of surface ozone monitoring sites analyzed in this study. We have gathered all available measurements in April and May from 1990 through 2012, including data from the monitoring networks operated by the U.S. National Park Service (NPS), the U.S. EPA's Clean Air Status and Trends Network (CASTNET), Air Quality System (AQS), and the NOAA Global Monitoring Division (GMD). These sites, located in western U.S. mountainous regions at ~1.2 to 3.5 km altitude, can intersect subsiding stratospheric ozone behind cold fronts more readily than lower-elevation sites.

Continuous hourly ozone measurements have been carried out at the sites only since the early 1990s. We focus our analysis on daily maximum 8-hour average ozone (hereafter MDA8), which usually includes the afternoon hours when the boundary layer is sufficiently deep to mix down ozone from aloft. The following biased measurements are excluded: anomalously low ozone at Lake Yellowstone and Rocky Mountain in 1990 that is inconsistent with other sites[63]**;** anomalously low ozone below 25 ppb for daily MDA8 values at Gothic during April 1-May 5, 2008 and at Pinedale during May 21-31, 2008; and anomalously low ozone at a few sites near the U.S.-Mexico border during spring 2012 that does not co-vary with other sites on synoptic time scales.

Representativeness of weekly ozonesonde measurements: Given the significant variability of tropospheric ozone mixing ratios on synoptic scales, we examine if the ozonesonde records with the weekly sampling frequency are suitable for assessing the actual year-to-year evolution of mean ozone levels above western North America. We compare observational records and model results co-sampled with observations in space and time with the 'true average' (i.e. continuous temporal sampling) determined from daily ozone fields archived from the model (**Figs.5-6 and Supplementary Fig.18**; orange circles vs. red lines).

Our analysis indicates that the signals of Pinatubo on UTLS ozone at Edmonton (**Fig.5**) and El Niño on UTLS ozone at Trinidad Head (**Fig.6b**) are fairly robust in their temporal evolution despite apparent differences in the detected signal due to the limited sampling from ozonesondes. At Boulder sonde site, inter-annual variability of UTLS ozone from the available weekly sampling is overall biased high during 1993-1997 and during 1999 compared to the model 'true average' determined from continuous temporal sampling (**Supplementary Figure 18b**; black and orange circles vs. red lines).

Ozonesonde profiles are available on fewer than 15% of days during April-May. Our comparison of the model co-sampled with the available sonde profiles versus the 'true' monthly model average indicates that the mid-tropospheric (~3-6 km altitude) ozonesonde records for April-May do not represent the actual inter-annual ozone variability in the free troposphere (**Supplementary Figure 18c-d**). For example, the sonde records do not clearly show the 2008 and 2011 high-ozone anomalies that are seen both in the model 'true' average and at the surface ozone record with continuous hourly measurements (**Fig.2**).

Satellite Observations: Satellite observations of total column ozone (**Supplementary Fig.12**) were obtained from NASA Goddard Space Flight Center. We use monthly mean Level-3 products from the Earth Probe Total Ozone Mapping Spectrometer (TOMS, 1997-2005)[64] and the Atmospheric Infrared Sounder (AIRS, 2003-2012)[65].

Supplementary Note 5: Additional Information for Model Experiments

Meteorology: All model simulations in the present study are nudged to NCEP-NCAR winds[66] using a pressure-dependent nudging technique (e.g., relaxing with a time scale of 6 hours in the surface level, ~60 hours by 100 hPa, and ~600 hours by 10 hPa)[67]. Our goal is to preserve the large-scale features of the observed airflow in the troposphere while allowing AM3 to simulate atmospheric circulation in the stratosphere. The weakening nudging strength in the UTLS minimizes the impacts of noise introduced via nudging. The global net stratosphere-to-troposphere flux of ozone in GFDL AM3 is ~535 Tg yr^{-1}, within the 400-600 Tg yr^{-1} range derived from tracer gas correlations observed in the lower stratosphere[68].

Emissions of ozone precursors: Anthropogenic emissions of aerosol and ozone precursors in both FIXEMIS and IAVFIRE simulations are set to the 1970-2010 climatology in order to isolate the role of meteorology alone. The CH_4 lower boundary condition for chemistry is held constant at 2000 levels. The

difference between IAVFIRE and FIXEMIS simulations (e.g. **Supplementary Fig.5a**) indicates the influence of inter-annual-varying fire emissions[69,70] relative to a climatology. We conduct a Background simulation, in which anthropogenic emissions are shut off over North America, but vary from year to year elsewhere based on historical emission inventories up to 2000 and the RCP8.5 projection beyond 2005 (refs. [71,72,73]).

Stratosphere-troposphere-aerosol chemistry: The GFDL AM3 model includes fully coupled stratosphere-troposphere chemistry and dynamics[74,75], thus allowing investigation of the interplay of processes influencing ozone from STT (e.g. circulation and abundance of ozone in the lower stratosphere, tropopause folding events and evolution in transit due to chemical and depositional losses). The tropospheric chemistry is based on a modified version of the chemical scheme in *Horowitz et al* [2003, 2007] as described by *Naik et al* [2013][74,76,77]. The stratospheric chemistry includes the full range of gas phase reactions covering the HO_x, NO_x, ClO_x, and BrO_x catalytic cycles and heterogeneous reactions on sulfate aerosols (liquid ternary H_2SO_4-HNO_3-H_2O solution) and polar stratospheric clouds[75,78].

The influence from major volcanic eruptions is imposed through the specification of monthly time series of zonal mean, multi-wavelength aerosol extinction as a function of altitude and latitude based on satellite measurements[79,80]. The spatial and temporal variability of stratospheric aerosol surface area density is calculated from the 1-micron constructed aerosol extinction using the relationships of *Thomason et al*[81]. The model captures observed decreasing trends in stratospheric ozone during 1960-2000 due to anthropogenic halocarbon emissions[75,82]. In the presence of anthropogenic chlorine, after the eruption of El Chichón (1982) and Mt. Pinatubo (1991), simulated chlorine radicals increased and the chlorine reservoirs decreased, leading to a pronounced ozone decrease in the lower stratosphere as seen in the observations[75].

Supplementary References

1 Langford, A. O., O'Leary, T. J., Masters, C. D., Aikin, K. C. & Proffitt, M. H. Modulation of middle and upper tropospheric ozone at northern midlatitudes by the El Nino Southern Oscillation. *Geophys. Res. Lett.* **25**, 2667-2670 (1998).

2 Langford, A. O. Stratosphere-troposphere exchange at the subtropical jet: contribution to the tropospheric ozone budget at midlatitudes. *Geophys. Res. Lett.* **26**, 2449-2452 (1999).

3 Zeng, G. & Pyle, J. A. Influence of El Nino Southern Oscillation on stratosphere/troposphere exchange and the global tropospheric ozone budget. *Geophys. Res. Lett.* **32**, L01814 (2005).

4 Tarasick, D. W., Fioletov, V. E., Wardle, D. I., Kerr, J. B. & Davies, J. Changes in the vertical distribution of ozone over Canada from ozonesondes: 1980-2001. *J. Geophys. Res.* **110**, D02304 (2005).

5 Thouret, V. *et al.* Tropopause referenced ozone climatology and inter-annual variability (1994-2003) from the

MOZAIC programme. *Atmospheric Chemistry and Physics* **6**, 1033-1051 (2006).
6 Ordonez, C. *et al.* Strong influence of lowermost stratospheric ozone on lower tropospheric background ozone changes over Europe. *Geophys. Res. Lett.* **34**, L07805 (2007).
7 Koumoutsaris, S., Bey, I., Generoso, S. & Thouret, V. Influence of El Nino-Southern Oscillation on the interannual variability of tropospheric ozone in the northern midlatitudes. *J. of Geophys. Res.* **113**, D19301 (2008).
8 Terao, Y., Logan, J. A., Douglass, A. R. & Stolarski, R. S. Contribution of stratospheric ozone to the interannual variability of tropospheric ozone in the northern extratropics. *J. Geophys. Res.* **113**, D18309 (2008).
9 Hsu, J. & Prather, M. J. Stratospheric variability and tropospheric ozone. *Journal of Geophysical Research-Atmospheres* **114**, D06102 (2009).
10 Voulgarakis, A., Hadjinicolaou, P. & Pyle, J. A. Increases in global tropospheric ozone following an El Nino event: examining stratospheric ozone variability as a potential driver. *Atmospheric Science Letters* **12**, 228-232 (2011).
11 Cuevas, E. *et al.* Assessment of atmospheric processes driving ozone variations in the subtropical North Atlantic free troposphere. *Atmospheric Chemistry and Physics* **13**, 1973-1998 (2013).
12 Pausata, F. S. R., Pozzoli, L., Vignati, E. & Dentener, F. J. North Atlantic Oscillation and tropospheric ozone variability in Europe: model analysis and measurements intercomparison. *Atmospheric Chemistry and Physics* **12**, 6357-6376 (2012).
13 Tang, Q., P. G. Hess, B. Brown-Steiner, and D. E. Kinnison. Tropospheric ozone decrease due to the Mount Pinatubo eruption: Reduced stratospheric influx. *Geophys. Res. Lett.* **40**, 5553-5558 (2013).
14 Neu, J. L. *et al.* Tropospheric ozone variations governed by changes in stratospheric circulation. *Nature Geoscience* **7**, 340-344 (2014).
15 Collins, W. J. *et al.* Effect of stratosphere-troposphere exchange on the future tropospheric ozone trend. *Journal of Geophysical Research-Atmospheres* **108**, 8528 (2003).
16 Stevenson, D. S. *et al.* Multimodel ensemble simulations of present-day and near-future tropospheric ozone. *Journal of Geophysical Research-Atmospheres* **111**, 23 (2006).
17 Hegglin, M. I. & Shepherd, T. G. Large climate-induced changes in ultraviolet index and stratosphere-to-troposphere ozone flux. *Nature Geoscience* **2**, 687-691 (2009).
18 Zeng, G., Morgenstern, O., Braesicke, P. & Pyle, J. A. Impact of stratospheric ozone recovery on tropospheric ozone and its budget. *Geophysical Research Letters* **37**, L09805 (2010).
19 Sprenger, M. & Wernli, H. A northern hemispheric climatology of cross-tropopause exchange for the ERA15 time period (1979-1993). *J. Geophys. Res.* **108**, 8521 (2003).
20 Hess, P. G. & Lamarque, J. F. Ozone source attribution and its modulation by the Arctic oscillation during the spring months. *J. Geophys. Res.* **112**, 1-17 (2007).
21 Hess, P. G. & Zbinden, R. Stratospheric impact on tropospheric ozone variability and trends: 1990-2009. *Atmos. Chem. Phys.* **13**, 649-674 (2013).
22 Stohl, A. *et al.* Stratosphere-troposphere exchange: A review, and what we have learned from STACCATO. *Journal of Geophysical Research-Atmospheres* **108**, (2003).
23 Doherty, R. M., Stevenson, D. S., Johnson, C. E., Collins, W. J. & Sanderson, M. G. Tropospheric ozone and El Nino-Southern Oscillation: Influence of atmospheric dynamics, biomass burning emissions, and future climate change. *J. Geophys. Res.* **111**, D19304 (2006).
24 Ziemke, J. R., Chandra, S., Oman, L. D. & Bhartia, P. K. A new ENSO index derived from satellite measurements of column ozone. *Atmospheric Chemistry and Physics* **10**, 3711-3721 (2010).
25 Škerlak, B., Sprenger, M. & Wernli, H. A global climatology of stratosphere-troposphere exchange using the ERA-Interim data set from 1979 to 2011. *Atmos. Chem. Phys.* **14**, 913-937 (2014).
26 Lefohn, A. S., Oltmans, S. J., Dann, T. & Singh, H. B. Present-day variability of background ozone in the lower troposphere. *J. Geophys. Res.* **106**, 9945-9958 (2001).
27 Langford, A. O., Aikin, K. C., Eubank, C. S. & Williams, E. J. Stratospheric contribution to high surface ozone in Colorado during springtime. *Geophys. Res. Lett.* **36**, L12801 (2009).
28 Langford, A. O. *et al.* Stratospheric influence on surface ozone in the Los Angeles area during late spring and early summer of 2010. *J. Geophys. Res.* **117**, D00v06 (2012).
29 Lin, M. *et al.* Springtime high surface ozone events over the western United States: Quantifying the role of stratospheric intrusions. *J. Geophys. Res.* **117**, D00V22 (2012).
30 Yates, E. L. *et al.* Airborne observations and modeling of springtime stratosphere-to-troposphere transport over California. *Atmos. Chem. Phys.* **13**, 12481-12494 (2013).
31 Ambrose, J. L., Reidmiller, D. R. & Jaffe, D. A. Causes of high O-3 in the lower free troposphere over the Pacific Northwest as observed at the Mt. Bachelor Observatory. *Atmospheric Environment* **45**, 5302-5315 (2011).
32 U.S. Environmental Protection Agency. Treatment of data influenced by exceptional events, Federal Register 72 (55),

13,560-13,581 (2007). Further details and updates available at http://www.epa.gov/ttn/analysis/exevents.htm.
33 State of Colorado. Technical support document for the May 24, 2010, stratospheric ozone intrusion exceptional event, report, Air Pollut. Control Div., Colo. Dep. of Public Health and Environ., Denver (2011).
34 State of Wyoming. Exceptional event demonstration package for the Environmental Protection Agency: South Pass, Wyoming Ozone Standard Exceedances on February 27–28, March 6–7, and March 10–13, 2009, report, Dep. of Environ. Qual., Cheyenne, Wyo (2011).
35 State of Wyoming. Exceptional Event Demonstration Package for the Environmental Protection Agency, Thunder Basin, Wyoming Ozone Standard Exceedance, June 6, 2012, http://deq.state.wy.us/aqd/Resources-Monitoring/Natural%20Exceptional%20Events/June_6_2012_SI_Package.pdf (2012).
36 State of Wyoming. Exceptional Event Demonstration Package for the Environmental Protection Agency, Big Piney and Boulder, Wyoming Ozone Standard Exceedances, June 14, 2012. Technical Demonstration: http://www.epa.gov/ttn/analysis/docs/June_14_2012_BigPiney_Boulder_SI_Package.pdf. EPA Response letter: http://www.epa.gov/ttn/analysis/docs/June_14_2012_Strat_O3_Concurrence_Letter_28_March_2014.pdf (2014).
37 Prather, M. CATASTROPHIC LOSS OF STRATOSPHERIC OZONE IN DENSE VOLCANIC CLOUDS. *Journal of Geophysical Research-Atmospheres* **97**, 10187-10191 (1992).
38 Fahey, D. W. *et al.* Insitu measurements constraining the role of sulfate aerosols in midlatitude ozone depletion. *Nature* **363**, 509-514 (1993).
39 McCormick, M. P., Thomason, L. W. & Trepte, C. R. Atmospheric effects of the Mt. Pinatubo eruption *Nature* **373**, 399-404 (1995).
40 Oltmans, S. J. *et al.* Trends of ozone in the troposphere. *Geophysical Research Letters* **25**, 139-142 (1998).
41 Fusco, A. C. & Logan, J. A. Analysis of 1970-1995 trends in tropospheric ozone at Northern Hemisphere midlatitudes with the GEOS-CHEM model. *Journal of Geophysical Research-Atmospheres* **108**, 4449 (2003).
42 Bronnimann, S. *et al.* Extreme climate of the global troposphere and stratosphere in 1940-42 related to El Nino. *Nature* **431**, 971-974 (2004).
43 Baldwin, M. P. & Osullivan, D. STRATOSPHERIC EFFECTS OF ENSO-RELATED TROPOSPHERIC CIRCULATION ANOMALIES. *Journal of Climate* **8**, 649-667 (1995).
44 Randel, W. J., Garcia, R. R., Calvo, N. & Marsh, D. ENSO influence on zonal mean temperature and ozone in the tropical lower stratosphere. *Geophys. Res. Lett.* **36**, L15822 (2009).
45 Calvo, N., Garcia, R. R., Randel, W. J. & Marsh, D. R. Dynamical Mechanism for the increase in tropical upwelling in the lowermost tropical stratosphere during warm ENSO events. *J. Atmos. Sci.* **67**, 2331-2340 (2010).
46 Garcia-Herrera, R., Calvo, N., Garcia, R. R. & Giorgetta, M. A. Propagation of ENSO temperature signals into the middle atmosphere: A comparison of two general circulation models and ERA-40 reanalysis data. *Journal of Geophysical Research-Atmospheres* **111**, D06101 (2006).
47 Manzini, E. Atmospheric Science: ENSO and the stratosphere. *Nature Geoscience* **2**, 749-750 (2009).
48 Li, Y. & Lau, N. C. Influences of ENSO on Stratospheric Variability, and the Descent of Stratospheric Perturbations into the Lower Troposphere. *J. Clim.* **26**, 4725-4748 (2013).
49 Stohl, A. *et al.* Stratosphere-troposphere exchange: A review, and what we have learned from STACCATO. *J. Geophys. Res.* **108**, 8516 (2003).
50 Danielsen, E. F. *et al.* Three-dimensional analysis of potential vorticity associated with tropopause folds and observed variations of ozone and carbon monoxide. *J. Geophys. Res.* **92**, 2103-2111 (1987).
51 Cooper, O. *et al.* On the life cycle of a stratospheric intrusion and its dispersion into polluted warm conveyor belts. *J. Geophys. Res.* **109**, D23S09 (2004).
52 Pan, L. L. *et al.* Chemical behavior of the tropopause observed during the Stratosphere-Troposphere Analyses of Regional Transport experiment. *J. Geophys. Res.* **112**, D18110 (2007).
53 Browell, E. V., Danielsen, E. F., Ismail, S., Gregory, G. L. & Beck, S. M. TROPOPAUSE FOLD STRUCTURE DETERMINED FROM AIRBORNE LIDAR AND INSITU MEASUREMENTS. *J. Geophys. Res.* **92**, 2112-2120 (1987).
54 Langford, A. O., Masters, C. D., Proffitt, M. H., Hsie, E. Y. & Tuck, A. F. Ozone measurements in a tropopause fold associated with a cut-off low system. *Geophys. Res. Lett.* **23**, 2501-2504 (1996).
55 Oltmans, S. J. *et al.* Summer and spring ozone profiles over the North Atlantic from ozonesonde measurements. *J. Geophys. Res.* **101**, 29179-29200 (1996).
56 Thompson, A. M. *et al.* Intercontinental Chemical Transport Experiment Ozonesonde Network Study (IONS) 2004: 2. Tropospheric ozone budgets and variability over northeastern North America. *J. Geophys. Res.* **112**, D12s13 (2007).
57 Trenberth, K. E. *et al.* Progress during TOGA in understanding and modeling global teleconnections associated with

tropical sea surface temperatures. *J. Geophys. Res.* **103**, 14291-14324 (1998).
58 National Oceanic and Atmospheric Administration-Climate Prediction Center. El Niño and La Niña related winter features over North America, available at http://www.cpc.ncep.noaa.gov/products/analysis_monitoring/ensocycle/nawinter.shtml (2005).
59 Li, Y. & Lau, N. C. Impact of ENSO on the Atmospheric Variability over the North Atlantic in Late Winter-Role of Transient Eddies. *J. Clim.* **25**, 320-342 (2012).
60 Seager, R. *et al.* Adjustment of the atmospheric circulation to tropical Pacific SST anomalies: Variability of transient eddy propagation in the Pacific-North America sector. *Q. J. R. Meteorol. Soc.* **136**, 277-296 (2010).
61 Appenzeller, C. & Holton, J. R. Seasonal variation of mass transport across the tropopause. *J. Geophys. Res.* **101**, 15071-15078 (1996).
62 Vallis, G. K. *Climate and the Oceans*. Pages 140-155 (Princeton University Press, 2012).
63 Jaffe, D. & Ray, J. Increase in surface ozone at rural sites in the western US. *Atmospheric Environment* **41**, 5452-5463 (2007).
64 TOMS Science Team,TOMS/Earth Probe Total Column Ozone Monthly L3 Global 1x1.25 deg Lat/Lon Grid,version 008, Greenbelt, MD: NASA Goddard Space Flight Center, Accessed October 2011 at http://disc.sci.gsfc.nasa.gov/datacollection/TOMSEPL3mtoz_V008.html
65 Susskind, J., Barnet, C. D. & Blaisdell, J. M. Retrieval of atmospheric and surface parameters from AIRS/AMSU/HSB data in the presence of clouds. *Ieee Transactions on Geoscience and Remote Sensing* **41**, 390-409 (2003).
66 Kalnay, E. *et al.* The NCEP/NCAR 40-year reanalysis project. *Bulletin of the American Meteorological Society* **77**, 437-471 (1996).
67 Lin, M. *et al.* Transport of Asian ozone pollution into surface air over the western United States in spring. *J. Geophys. Res.* **117**, D00V07 (2012).
68 McLinden, C. A. *et al.* Stratospheric ozone in 3-D models: A simple chemistry and the cross-tropopause flux. *J. Geophys. Res.* **105**, 14653-14665 (2000).
69 Schultz, M. G. *et al.* Global wildland fire emissions from 1960 to 2000. *Global Biogeochemical Cycles* **22**, Gb2002 (2008).
70 van der Werf, G. R. *et al.* Global fire emissions and the contribution of deforestation, savanna, forest, agricultural, and peat fires (1997-2009). *Atmospheric Chemistry and Physics* **10**, 11707-11735 (2010).
71 Lamarque, J. F. *et al.* Historical (1850-2000) gridded anthropogenic and biomass burning emissions of reactive gases and aerosols: methodology and application. *Atmospheric Chemistry and Physics* **10**, 7017-7039 (2010).
72 Moss, R. H. *et al.* The next generation of scenarios for climate change research and assessment. *Nature* **463**, 747-756 (2010).
73 Riahi, K. *et al.* RCP-8.5: exploring the consequence of high emission trajectories. Climatic Change. *Climate Change* (2011).
74 Naik, V. *et al.* Impact of preindustrial to present-day changes in short-lived pollutant emissions on atmospheric composition and climate forcing. *J. Geophys. Res.* **118**, 8086-8110 (2013).
75 Austin, J., Horowitz, L. W., Schwarzkopf, M. D., Wilson, R. J. & Levy, H. Stratospheric Ozone and Temperature Simulated from the Preindustrial Era to the Present Day. *J. Clim.* **26**, 3528-3543 (2013).
76 Horowitz, L. W. *et al.* A global simulation of tropospheric ozone and related tracers: Description and evaluation of MOZART, version 2. *J. Geophys. Res.* **108**, 4784 (2003).
77 Horowitz, L. W. *et al.* Observational constraints on the chemistry of isoprene nitrates over the eastern United States. *J. Geophys. Res.* **112**, D12s08 (2007).
78 Austin, J. & Wilson, R. J. Sensitivity of polar ozone to sea surface temperatures and halogen amounts. *J. Geophys. Res.* **115**, D18303 (2010).
79 Stenchikov, G. L. *et al.* Radiative forcing from the 1991 Mount Pinatubo volcanic eruption. *Journal of Geophysical Research-Atmospheres* **103**, 13837-13857 (1998).
80 Stenchikov, G. *et al.* Arctic Oscillation response to volcanic eruptions in the IPCC AR4 climate models. *Journal of Geophysical Research-Atmospheres* **111**, D07107 (2006).
81 Thomason, L. W., Poole, L. R. & Deshler, T. A global climatology of stratospheric aerosol surface area density deduced from stratospheric aerosol and gas experiment II measurements: 1984-1994. *Journal of Geophysical Research-Atmospheres* **102**, 8967-8976 (1997).
82 Donner, L. J. *et al.* The Dynamical Core, Physical Parameterizations, and Basic Simulation Characteristics of the Atmospheric Component AM3 of the GFDL Global Coupled Model CM3. *J. Clim.* **24**, 3484-3519 (2011).
